# Uric Acid Variability Is Associated with Poor Prognosis in Heart Failure

**DOI:** 10.3390/jcm15062330

**Published:** 2026-03-18

**Authors:** Viana Copeland, Shir Elimeleh, Assi Milwidsky, Noam Makmal, Ranel Loutati, Boris Fishman, Yishay Wasserstrum, Moti Zwilling, Elad Maor, Ehud Grossman

**Affiliations:** 1 The Olga and Lev Leviev Heart Center, Sheba Medical Center, Ramat Gan 52621, Israel; 2 The Faculty of Medical and Health Sciences, Tel-Aviv University, Tel Aviv 69978, Israel; 3 Division of Cardiology, Department of Medicine, Columbia University Irving Medical Center, New York, NY 10032, USA; 4 The Department of Economics and Business Administration, Ariel University, Ariel 40700, Israel; 5 The Adelson School of Medicine, Ariel University, Ariel 40700, Israel; ehudgrossman@gmail.com

**Keywords:** uric acid, heart failure, prognosis, risk stratification

## Abstract

**Aims:** Elevated uric acid (UA) levels correlate with worse heart failure (HF) outcomes, but past studies used single UA measurements. The effect of intra-individual UA fluctuations, beyond mean levels, is unclear. This study assesses the relationship between serum UA variability and adverse clinical outcomes in HF patients. **Methods**: We analyzed 18,115 HF patients from the SHEBAHEART registry (2009–2025) with at least three UA measurements within three years of diagnosis. UA variability was quantified as the mean deviation (MD) from each patient’s average UA level and divided into quartiles: Q1 (≤−0.69 mg/dL), Q2–Q3 (>−0.69 and <1.53 mg/dL, reference), and Q4 (≥1.53 mg/dL). All-cause mortality was the primary outcome and HF hospitalization was secondary. Cox regression, propensity score matching, and subgroup analyses were used. **Results**: Over a median follow-up of 4.3 years (IQR 1.6–7.7), 36% of patients were hospitalized for HF and 65.5% died. UA variability showed a graded association with outcomes. Low variability (Q1) was linked to reduced mortality (HR 0.79, 95% CI 0.75–0.83) and HF hospitalization (HR 0.84, 95% CI 0.79–0.90), while high variability (Q4) increased mortality (HR 1.58, 95% CI 1.51–1.69) and hospitalization risk (HR 1.17, 95% CI 1.10–1.25) (all *p* < 0.001). These associations remained after propensity score matching and across HF subgroups. **Conclusions**: UA variability is a robust, independent predictor of mortality and HF hospitalization. Serial UA monitoring may enhance risk stratification in HF management.

## 1. Introduction

Heart failure (HF) is a leading cause of morbidity and mortality worldwide, with outcomes shaped not only by established risk factors but also by metabolic and inflammatory pathways [1]. Among these, uric acid (UA) has emerged as a potential biomarker of cardiovascular risk [2,3]. Elevated serum UA levels are common in HF, driven by impaired renal clearance, tissue hypoxia, and xanthine oxidase activation, and are consistently associated with adverse prognosis, including increased mortality and re-hospitalization. Uric acid contributes to cardiovascular injury through oxidative stress, endothelial dysfunction, and inflammation, central mechanisms in HF, and higher levels are consistently linked to adverse cardiovascular outcomes.

This concept is supported by evidence linking uric acid to cardiovascular risk in hypertension and related phenotypes [4,5,6,7]. In recent years, the variability in biomarkers over time, which reflects within-patient biomarker fluctuations, has emerged as an important prognostic feature, complementing static baseline measurements. Greater intra-individual fluctuations in biomarkers such as blood pressure, cholesterol, and glucose have been shown to reflect systemic instability and to predict adverse cardiovascular outcomes independently of average levels [8,9,10,11,12,13]. In the context of HF, where disease trajectory is characterized by recurrent decompensations, hemodynamic shifts, and metabolic stress, UA variability may similarly provide insight into pathophysiologic instability beyond absolute concentrations [14,15,16].

Despite these considerations, the prognostic relevance of UA variability in HF remains poorly defined. Cardiovascular risk may depend not only on absolute uric acid levels but also on within-patient variability, reflecting fluctuations in congestion, renal function, neurohormonal activation, and treatment intensity.

Most studies have focused on single time-point measurements or the presence of hyperuricemia, without accounting for longitudinal changes [17]. Whether intra-individual fluctuations in UA amplify risk independent of mean levels, and whether such variability differs in impact across HF subtypes, is unknown. Accordingly, we sought to investigate the association between UA variability and all-cause mortality and rate of HF hospitalizations in a large, contemporary cohort of patients with HF.

## 2. Methods

### 2.1. Study Population

The study included all patients aged 18 years or older with a validated diagnosis of HF and at least three serum uric acid measurements obtained during a three-year follow-up period. Patients were excluded if they were younger than 18 years, lacked validation of the HF diagnosis, had fewer than three uric acid measurements, or had missing data in the population registry. The retrospective cohort included 18,115 consecutive adult patients with HF evaluated at Sheba Medical Center between January 2009 and February 2025. The analysis was conducted using the SHEBAHEART big data registry [18]. The study was approved by the Institutional Review Board, which granted a waiver of informed consent due to the retrospective nature of the analysis and the strict use of anonymized data. The primary outcome was all-cause mortality, retrieved from the Israeli Ministry of Interior through to February 2025. The secondary outcome was HF hospitalizations through to February 2025.

### 2.2. HF Definition

Diagnoses were established in accordance with the European Society of Cardiology (ESC) HF guidelines. Within the SHEBAHEART registry, HF diagnosis was established through a validated clinical process that included board-certified cardiologist assessment, review of hospitalization and outpatient records, and echocardiographic evaluation, with ICD-9 codes used only as supportive administrative identifiers [18]. International Classification of Diseases, Ninth Revision (ICD-9) codes 404.X and 428.X were used as supportive administrative identifiers.

### 2.3. HF Hospitalizations

HF hospitalization was defined using an integrated algorithm incorporating diagnostic, therapeutic, laboratory, and imaging criteria. Eligible events required a documented hospital admission and discharge with evidence of HF decompensation, including a discharge diagnosis of HF, cardiogenic shock, or pulmonary edema. Supportive criteria included intensification of diuretic therapy (initiation of intravenous furosemide or escalation in dose, timing, or route compared with baseline), initiation or escalation of vasoactive or vasodilator therapy, and laboratory worsening indicated by elevated inpatient BNP relative to baseline. Echocardiographic deterioration, defined as reduced left ventricular ejection fraction or increased inferior vena cava diameter, further supported HF hospitalization. Non-HF hospitalizations were excluded [19,20,21].

### 2.4. Study Variables and Outcomes

Baseline variables included age, sex, comorbidities, drug treatment, and laboratory and imaging parameters. HTN, DM, and other comorbidities were defined using ICD codes, laboratory thresholds, and chronic medication use recorded in the electronic medical record. Advanced chronic kidney disease (ACKD) was defined as a estimated glomerular filtration rate (eGFR) of ≤30 mL/min/1.73 m^2^ using CKD-EPI. Patients were further classified based on age groups, with patients ≥ 80 being classified as octogenarians or older and <80 as non-octogenarians. Anemia was defined as hemoglobin < 13 g/dL in men and <12 g/dL in women. History of use of urate-lowering therapy was captured and evaluated in subgroup analyses. Right ventricular function was assessed using tricuspid annular plane systolic excursion (TAPSE).

### 2.5. Definition and Calculation of UA Variability

Serum UA (mg/dL) measurements obtained during routine inpatient and outpatient care within three years of HF diagnosis were used to characterize absolute levels and intra-individual variability. To minimize reverse causality, follow-up was anchored at the first UA measurement after HF diagnosis. Same-day duplicate measurements were collapsed to one value per patient to limit surveillance bias. Variability was quantified as the mean deviation (MD), calculated as the average absolute difference between each UA measurement and the patient-specific mean UA value. The MD was calculated as the Σ of all absolute differences divided by the number of measurements (MD = (1/*n*) × Σ *|UA_i* − *UA_mean*|). This calculation captured the magnitude of fluctuations independent of direction. Quartiles of MD were used to allow non-linear risk assessment and to harmonize with prior variability studies, with the middle quartiles (Q2–Q3) being combined as a clinically interpretable ‘intermediate stability’ reference. Patients were stratified into quartiles of variability based on MD: Q1 (low variability, ≤−0.69 mg/dL), Q2–Q3 (mid-range, >−0.69 and <1.53 mg/dL; reference group), and Q4 (high variability, ≥1.53 mg/dL), allowing evaluation of risks associated with both low and high variability relative to a stable mid-range. Middle quartiles were combined to serve as a stable reference group, consistent with prior biomarker variability studies and to improve interpretability. Median UA variability delta (Δ) (mg/dL) was defined as the median within-patient change in UA variability relative to the overall cohort median UA variability across the 3-year observation period. To capture directional changes, UA trajectory was separately analyzed and categorized as increasing, stable, or declining based on the slope of serial measurements from linear regression.

### 2.6. Statistical Analysis

Follow-up time was calculated from the date of the first UA measurement after HF diagnosis to ensure temporal alignment between exposure assessment and outcome risk. We employed Cox proportional hazards models with follow-up time in years as the time scale. Categorical variables were compared across the three groups using chi-square tests or Fisher’s exact test, and continuous variables were analyzed using one-way ANOVA or the Kruskal−Wallis test. Based on the observed event rate and sample size (*n* = 18,115), the study demonstrated > 90% statistical power to detect the observed hazard ratios for the primary outcome at a two-sided alpha level of 0.05. Proportional hazards assumptions were evaluated graphically and using Schoenfeld residuals. Unadjusted and multivariable Cox models were constructed using covariates selected based on clinical relevance, literature, and statical significance (*p* < 0.05). Multivariate analysis consisted of both continuous covariates and binary variables including age, LVEF ≤ 40%, SPAP ≥ 40 mmHg, eGFR ≤ 30 mL/min/1.73 m^2^, BMI ≥ 30 kg/m^2^, baseline UA levels, and GDMT. Variables with more than 40% missing data were not included in the analysis to minimize bias. For variables with less missing data, complete-case analysis was performed, given the large sample size and to preserve interpretability.

Sensitivity analyses assessed robustness across HF subtype, age, sex, diuretic use that may influence uric acid levels including allopurinol or febuxostat, and major comorbidities that may affect mortality, and were repeated in both unmatched Cox models and propensity score-matched (PSM) cohorts. PSM was used as a sensitivity analysis to reduce residual confounding, using 1:2:1 nearest-neighbor matching without replacement (caliper 0.2 SD) for pairwise contrasts (Q1 vs. Q2–Q3 and Q4 vs. Q2–Q3). All results are presented as hazard ratios with 95% confidence intervals using R (version 4.5.0, R Foundation for Statistical Computing). The data that support the findings of this study are derived from the SHEBAHEART registry and are not publicly available due to privacy and institutional governance restrictions. De-identified data may be made available upon reasonable request and subject to institutional approvals and a data-sharing agreement [18]. During the preparation of this manuscript, generative artificial intelligence tools (ChatGPT, OpenAI, and Grok) were used for language refinement and editorial assistance.

## 3. Results

### 3.1. Baseline Characteristics

The cohort included 18,115 patients stratified by UA variability: UA variability was categorized into low (≤−0.69 mg/dL; *n* = 4529), mid-range (>−0.69 and <1.53 mg/dL; *n* = 9057), and high (≥1.53 mg/dL; *n* = 4529) based on distance cutoff values. Median age was slightly higher in the high-variability group compared with low- and mid-variability groups (Table 1). Patients with high variability had more advanced renal dysfunction (median eGFR 54 vs. 71 mL/min/1.73 m^2^, *p* < 0.001) and higher SPAP (44 vs. 39 mmHg, *p* < 0.001) compared with the low-variability group (Table 1).

After PSM (*n* = 8384), UA variability was categorized into low (*n* = 2096), mid-range (*n* = 4192), and high (*n* = 2096). Baseline distributions of sex and comorbidities including CVA did not differ between the groups (*p* = 0.824 and *p* = 0.180, respectively). Renal dysfunction, SPAP, and anemia prevalence were more pronounced in the high-variability group (Table 2). Furthermore, HFpEF was more prevalent in the high-variability group (Table 2).

### 3.2. Mortality and HF Hospitalization

During a median follow-up of 4.3 years (IQR 1.6–7.7), 11,872 of 18,115 patients (65.5%) died and 6535 (36%) were hospitalized for HF. Mortality increased stepwise across variability quartiles: 52.5% (2381) in Q1, 64% (5775) in Q2–Q3, and 82% (3716) in Q4. One-year mortality was 14% (649) in Q1, 17% (1557) in Q2–Q3, and 29% (1333) in Q4. HF hospitalization followed a similar gradient: 24.0% (1084) in Q1, 38% (3434) in Q2–Q3, and 44.5% (2017) in Q4 (Supplementary Figure S1). The one-year HF hospitalization rate was 15% overall (2690), ranging from 8% (349) in Q1 to 19% (879) in Q4.

**Figure 1:**
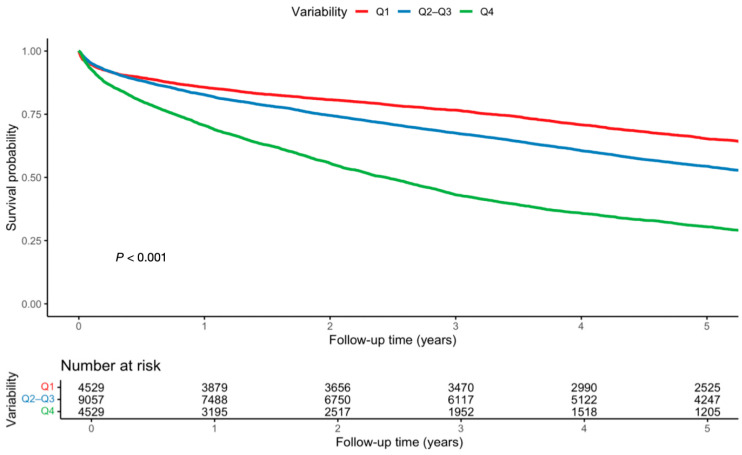
Overall survival by UA variability- Kaplan−Meier curves for all-cause mortality stratified by UA variability. Low variability was associated with improved survival, while high variability predicted excess mortality (log-rank *p* < 0.0001).

**Figure 2:**
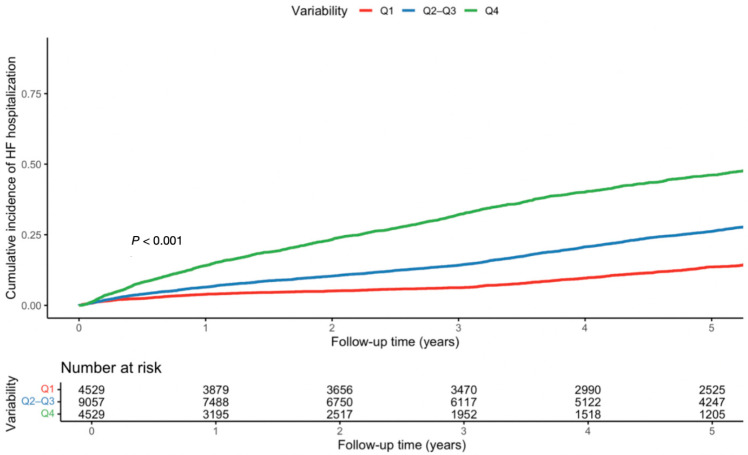
Cumulative HF-related hospitalizations based on UA variability—cumulative incidence functions for first HF-related hospitalization with death treated as a competing event, stratified by UA variability quartiles (Q1, Q2–Q3, Q4).

Low variability was associated with reduced mortality (HR 0.79, 95% CI; 0.75–0.83, *p* < 0.001), while high variability was associated with increased mortality (HR 1.59, 95% CI; 1.52–1.66, *p* < 0.001) (Table 3, Figure 1, Supplementary Tables S1 and S2). Findings persisted post PSM (Supplementary Tables S3–S5). For HF hospitalizations, low variability was related to reduced risk (HR 0.85, 95% CI; 0.80–0.90, *p* < 0.001), whereas high variability increased it (HR 1.18, 95% CI; 1.11–1.25, *p* < 0.001) (Table 4, Figure 2, Supplementary Tables S6 and S7). Findings persisted post PSM (Supplementary Tables S8 and S9).

### 3.3. Mortality—Subgroup Analysis

**Figure 3:**
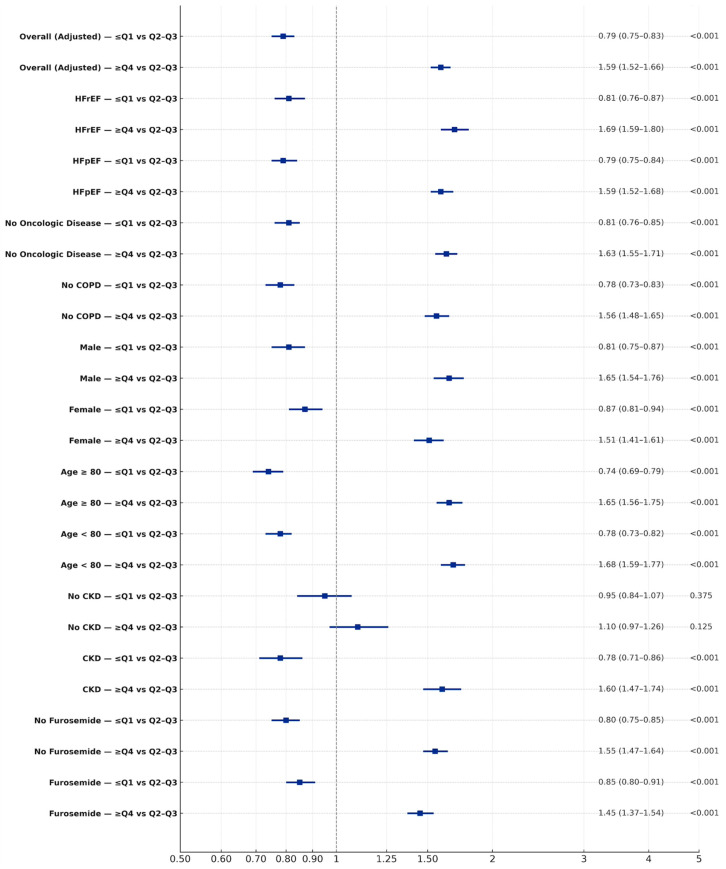
Adjusted hazard ratios for all-cause mortality by UA variability (subgroup analysis) pre PSM—adjusted Cox models comparing ≤ Q1 vs. Q2-Q3 and ≥Q4 vs. Q2-Q3 UA variability evaluating mortality.

**Figure 4:**
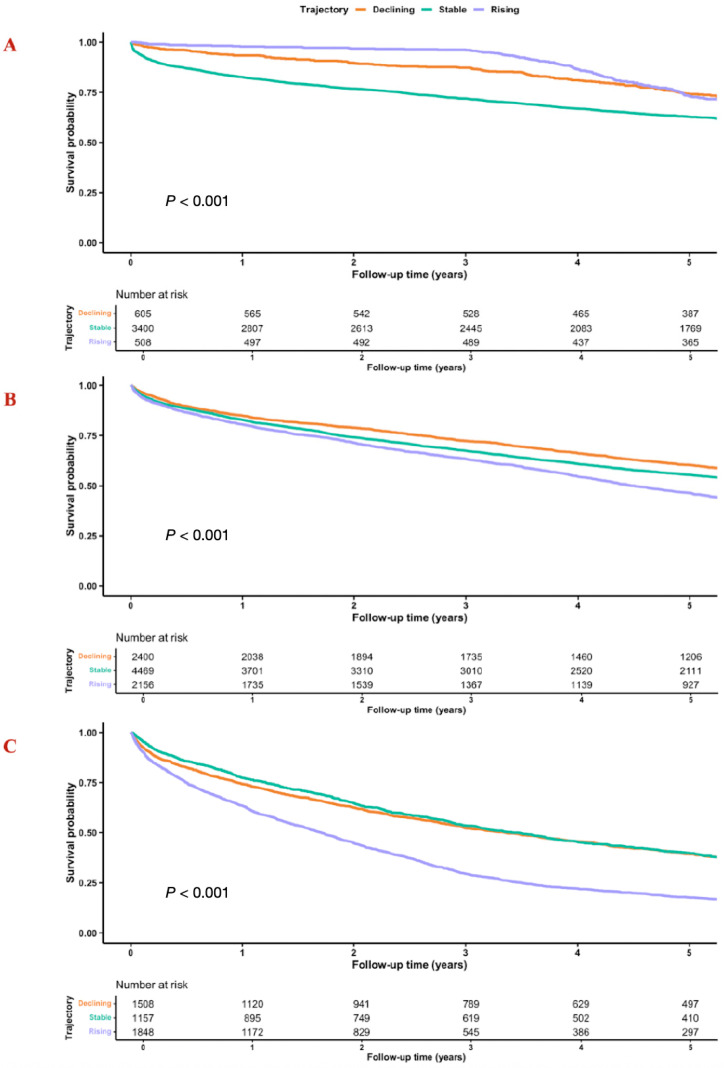
Survival by uric acid (UA) trajectory within variability strata. Kaplan–Meier curves for all-cause mortality illustrate how longitudinal UA trajectories interact with variability level. Within each variability quartile: low (Q1), intermediate (Q2–Q3), and high (Q4), patients were further stratified by trajectory pattern (declining, stable, or rising). (**A**) Low UA variability (Q1): Kaplan–Meier curves showing survival according to UA trajectory patterns (declining, stable, rising) among patients in the lowest UA variability quartile. (**B**) Intermediate UA variability (Q2–Q3): Kaplan–Meier curves showing survival according to UA trajectory patterns among patients with intermediate UA variability (reference quartiles). (**C**) High UA variability (Q4): Kaplan–Meier curves showing survival according to UA trajectory patterns among patients in the highest UA variability quartile.

Subgroup analyses confirmed the robustness of the findings. Low UA variability was associated with lower mortality in both HFpEF and HFrEF, whereas high variability was consistently linked to higher risk (Figure 3, Supplementary Figure S2). High variability patterns persisted post PSM. Comparable patterns were seen across sex and age groups (Figure 3). Similar results were observed after excluding patients with comorbidities that may affect survival such as oncologic disease, COPD, or end-stage renal disease (Figure 3). Furthermore, this trend persisted once adjusted for TAPSE. Among patients receiving furosemide that may affect uric acid levels [N = 11,957 (67%)], low variability was associated with reduced mortality and high variability with increased mortality (Figure 3, Supplementary Figure S3). In patients not receiving furosemide, high variability was further significantly associated with increased mortality (Figure 3). Among patients treated with uric-acid-lowering agents [N = 1315 (7.5%)], high variability was associated with significantly higher mortality (HR 1.49, 95% CI 1.03–2.14; *p* = 0.029). Finally, analyses of UA trajectories showed that stable or declining trajectories were consistently associated with favorable outcomes, whereas rising or unstable trajectories conferred greater risk, with the highest mortality rate observed among patients with both high variability and rising UA (HR 1.71, 95% CI; 1.57–1.86; *p* < 0.001) (Figure 4). These patterns were replicated in PSM analysis and persisted post matching (Supplementary Tables S10–S40).

### 3.4. Hospitalization—Subgroup Analysis

**Figure 5:**
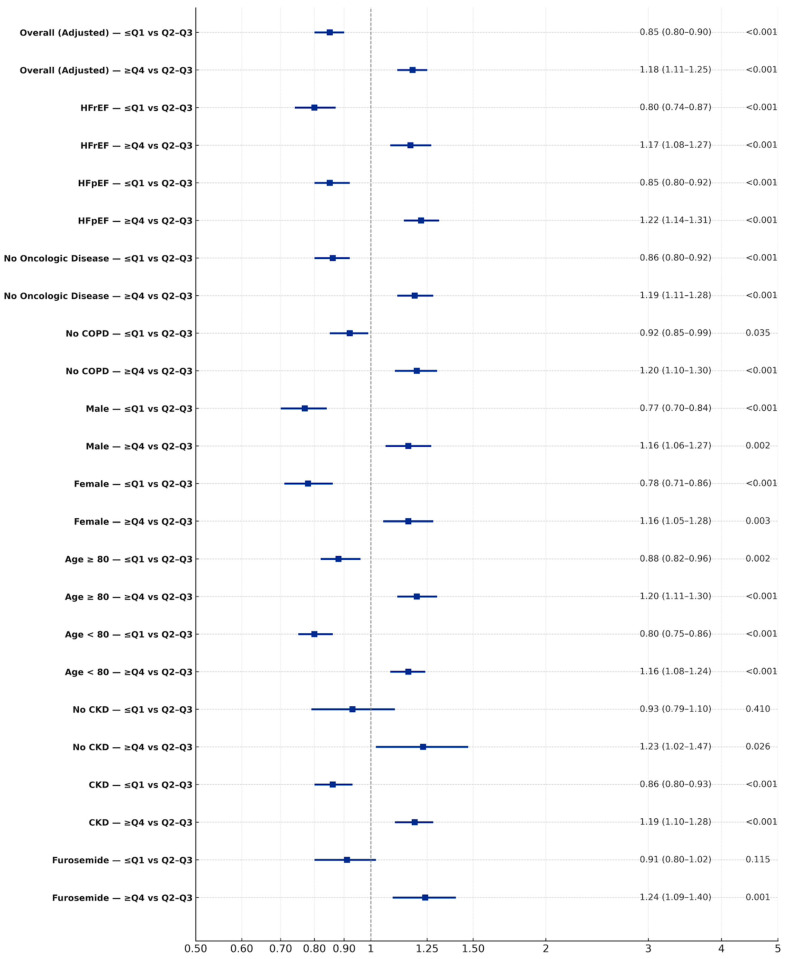
Adjusted hazard ratios for HF-related hospitalizations by UA variability (subgroup analysis) pre PSM—adjusted Cox models comparing ≤ Q1 vs. Q2-Q3 and ≥Q4 vs. Q2-Q3 UA variability.

Subgroup analyses confirmed the robustness of the findings. Low UA variability was consistently associated with fewer HF hospitalizations, whereas high variability was associated with higher risk across both HFpEF and HFrEF. High variability patterns persisted post PSM. Similar patterns were observed independent of age group and sex (Figure 5). Among patients receiving furosemide, low variability was associated with reduced hospitalizations and high variability with increased hospitalizations (Figure 5). In the absence of furosemide, high variability was further significantly associated with increased hospitalizations (Figure 5). Among patients treated with uric-acid-lowering agents, UA variability was not associated with HF hospitalization. These findings persisted post PSM. A similar trend was noted once adjusted for TAPSE. Finally, analyses of UA trajectories demonstrated that descending or stable trajectories were associated with lower rates of HF-related hospitalizations, particularly in patients with low or moderate variability, whereas rising UA trajectories were linked to higher risk. The greatest risk was observed in those with both high variability and rising UA levels (HR 1.15, 95% CI 1.02–1.31; *p* = 0.022) (Supplementary Tables S41–S71).

## 4. Discussion

In this large, contemporary cohort of HF patients, UA variability was a strong and independent predictor of mortality. Whereas prior work has largely focused on absolute UA concentrations, our findings highlight that the stability of UA values over time carries prognostic weight [22]. Patients with consistently low variability had better outcomes, while those with unstable patterns experienced substantially higher mortality, regardless of mean UA levels. Importantly, these associations extended beyond mortality to include HF hospitalizations, which followed a nearly identical graded relationship with UA variability.

Several mechanisms may underlie this association. UA, the end product of purine metabolism, is closely linked to xanthine oxidase activity, oxidative stress, endothelial dysfunction, and inflammation, key drivers of HF progression. Fluctuations in UA may reflect oscillatory oxidative stress, intermittent renal dysfunction, recurrent congestion–decongestion cycles, or variability in diuretic exposure, thereby signaling hemodynamic and metabolic instability. Unlike chronically elevated UA, which may indicate sustained risk, increased variability may capture episodes of acute decompensation and unstable renal handling of urates. The persistence of these associations in metabolic subgroups such as obese and diabetic patients further suggests that UA variability integrates shared metabolic, inflammatory, and renal stress pathways contributing to adverse cardiovascular outcomes [2,22,23,24,25].

The consistent associations across subgroups strengthen the generalizability of our findings. Both HFpEF and HFrEF patients demonstrated the same pattern, suggesting that UA variability reflects a pathway that cuts across ejection fraction phenotypes. This is particularly relevant in HFpEF, where traditional biomarkers and therapies have limited discriminatory value [3,26,27,28]. Importantly, this association persisted in patients not treated with furosemide, a drug known to increase UA levels [29,30]. This suggests that variability is not simply a pharmacologic artifact of loop diuretic use, but rather a more fundamental reflection of patient-level instability.

Trajectory analyses further extend this concept. Not only did the magnitude of variability matter, but the trajectory itself was informative. Patients with stable or slightly declining UA had substantially better outcomes, while those with highly unstable or rising trajectories carried the highest risk [2,22]. This suggests that serial monitoring of biomarker trajectory, not just point estimates, may be central to understanding risk in chronic HF [22].

UA variability was strongly associated with HF hospitalizations. Low variability was consistently linked to lower hospitalization risk, whereas high variability conferred increased risk, independent of HF subtype and major clinical factors. These findings indicate that UA instability predicts not only mortality but also recurrent decompensation events that drive morbidity and healthcare utilization [8,9,10,11,12]. The effect appeared more pronounced for mortality in older adults, while in younger patients both mortality and hospitalization were significantly impacted. Importantly, these associations persisted after adjustment for baseline UA levels, underscoring the incremental prognostic value of variability beyond static measurements [2,22].

Clinically, UA variability may enhance risk stratification and it supports the value of serial monitoring rather than single measurements [2,21]. Whether UA variability is a causal mediator or simply a marker of metabolic and hemodynamic instability remains uncertain. If causal, stabilizing UA levels could improve outcomes; if not, variability may serve primarily as a prognostic indicator rather than a therapeutic target. Prior UA-lowering trials in HF have focused on absolute reduction rather than stabilization, highlighting the need for future studies to clarify this distinction [22].

Finally, our results contribute to the broader understanding of biomarker variability as a clinical entity. Whereas prior studies have focused predominantly on single baseline uric acid levels, intra-individual longitudinal variability has not been rigorously examined as an independent prognostic marker after adjustment and propensity matching [3,31,32,33,34]. Similar to variability in glucose, blood pressure, and cholesterol, each of which has emerged as an independent predictor of outcomes in HF patients, UA variability appears to carry unique prognostic information [8,9,10,11,12,22]. If validated prospectively, UA variability could serve as a readily available, low-cost biomarker for refining management strategies in HF, where prognostic tools remain limited.

## 5. Strengths and Limitations

As a single-center study from a tertiary care facility, our findings may have limited generalizability to diverse populations with different dietary or healthcare access profiles. Nonetheless, it is important to note that the Israeli population is very diverse and shares a common genetic ancestry with many other populations, thus aiding generalizability [35]. Nonetheless, single-center registry studies may limit generalizability and introduce referral bias [36]. Because UA testing frequency was determined by clinical care, residual surveillance bias cannot be fully excluded, although consistent results across multiple sensitivity analyses mitigate this concern. Unmeasured confounders, such as a purine-rich diet, xanthine oxidase activity, or socioeconomic factors, could influence UA variability and outcomes. New York Heart Association class is inconsistently documented in structured fields and therefore could not be incorporated as a primary covariate without introducing differential missingness. Analytical variation in UA assays, due to laboratory techniques or timing of blood draws, may contribute to observed variability, though standardized protocols at our institution minimized this risk. Future studies should validate findings using uniform measurement schedules. However_,_ this study has key strengths, including its large, contemporary cohort with long follow-up, use of serial UA measurements to assess variability and trajectory, and consistent associations across mortality and HF hospitalizations independent of mean levels. Robust methodology with multivariable models, PSM, and subgroup and sensitivity analyses, further support the findings, while the use of national mortality data and routine laboratory tests enhances reliability and clinical relevance.

## 6. Conclusions

Patients with stable UA levels had substantially lower risk, while those with unstable or rising trajectories experienced markedly higher mortality and HF hospitalizations. Hospitalization analyses confirmed that UA variability captures not only long-term prognosis but also short-term vulnerability to acute decompensation. Taken together, these findings challenge reliance on single-point UA measurements and underscore the clinical importance of longitudinal biomarker monitoring. UA variability may integrate hemodynamic burden, renal function, and metabolic resilience into a single measurable parameter. Its low cost and widespread availability make it attractive for routine clinical use. Future prospective and interventional studies should determine whether therapeutic stabilization of UA fluctuations can mitigate risk and improve both survival and hospitalization outcomes in HF.

## Figures and Tables

**Table 1:** Baseline characteristics by UA variability before matching: Comparison of demographics, comorbidities, medications, echocardiographic findings, and UA-related measures across Q1, Q2–Q3, and Q4 groups prior to adjustment.

	UA Variability						
Variable	Overall (N = 18,115)	Low (Q1) (N = 4529)	Mid (Q2–Q3) (N = 9057)	High (Q4) (N = 4529)	*p*-Value	ANOVA Statistic	Chi-Square Statistic
***General characteristics***							
**Age (Y)**	74 [64, 82]	73 [63, 81]	73 [64, 82]	75 [65, 83]	**<0.001**	F = 74.4	
**Male (%)**	11,141 (61%)	2765 (61%)	5632 (62%)	2744 (61%)	0.376		X^2^ = 0.945
***Comorbidities***							
**ACKD (%)**	1909 (10%)	234 (5%)	906 (10%)	769 (17%)	**<0.001**		X^2^ = 249.47
**HTN (%)**	12,626 (70%)	2924 (65%)	6462 (71%)	3240 (71%)	**<0.001**		X^2^ = 219.56
**DM (%)**	10,478 (58%)	2321 (51%)	5346 (59%)	2811 (62%)	**<0.001**		X^2^ = 181.81
**AF (%)**	9206 (51%)	2013 (44%)	4642 (51%)	2551 (56%)	**<0.001**		X^2^ = 7.78
**CVA (%)**	4303 (24%)	922 (20%)	2241 (25%)	1140 (25%)	**<0.001**		X^2^ = 51.27
**COPD (%)**	2798 (15%)	520 (11%)	1417 (16%)	861 (19%)	**<0.001**		X^2^ = 16.84
**IHD (%)**	9588 (53%)	2209 (49%)	4911 (54%)	2468 (54%)	**<0.001**		X^2^ = 43.19
**Anemia (%)**	11,270 (62%)	2500 (55%)	5581 (62%)	3189 (70%)	**<0.001**		X^2^ = 322.39
***Medications***							
**RAASi (%)**	10,129 (57%)	2526 (57%)	5083 (57%)	2553 (57%)	0.998		X^2^ = 0.11
**BB (%)**	11,561 (65%)	2893 (65%)	5754 (64%)	2914 (65%)	0.382		X^2^ = 0.77
**CCB (%)**	5302 (30%)	1367 (30%)	2629 (29%)	1306 (29%)	0.446		X^2^ = 0.96
**Furosemide (%)**	11,957 (67%)	3020 (67%)	5931 (66%)	3006 (67%)	0.364		X^2^ = 0.92
**Thiazides (%)**	1620 (9%)	324 (7%)	854 (9%)	442 (10%)	**<0.001**		X^2^ = 32.4
**MRA (%)**	3858 (21%)	942 (21%)	1960 (22)	956 (21%)	0.264		X^2^ = 0.72
**SGLT2i (%)**	1215 (7%)	328 (7%)	605 (7%)	282 (7%)	0.998		X^2^ = 0.48
**UA-lowering medications (%)**	1315 (7%)	310 (6.5%)	595 (6.5%)	410 (9%)	**<0.001**		X^2^ = 7.64
***Lab tests***							
**eGFR mL/min/1.73 m^2^**	63 [43, 85]	71 [50, 90]	64 [44, 86]	54 [34, 76]	**<0.001**	F = 124.01	
***Echocardiographic parameters***							
**LVEF (%)**	50 [30, 60]	50 [33, 60]	52 [33, 60]	44 [30, 60]	**<0.001**	F = 487.85	
**HFpEF (%)**	8515 (47%)	1927 (42.5%)	4547 (50%)	2041 (45%)	**<0.001**		X^2^ = 151.67
**SPAP (mmHg)**	41 [33, 51]	39 [31, 49]	40 [33, 50]	44 [35, 54]	**<0.001**	F = 487.85	
**LVEDD (mm)**	4.9 [4.4, 5.5]	4.9 [4.4, 5.5]	4.9 [4.4, 5.4]	5.0 [4.5, 5.6]	**<0.001**	F = 68.59	
**TAPSE (cm)**	1.8 [1.4, 2.1]	1.8 [1.5, 2.1]	1.8 [1.5, 2.2]	1.6 [1.3, 2.0]	**0.020**	F = 46.44	
***UA-related parameters***							
**Baseline First UA mg/dL**	6.6 [5.2, 8.4]	6.0 [4.7, 7.6]	6.5 [5.2, 8.1]	7.7 [6.2, 9.6]	**<0.001**	F = 48.73	
**Median UA variability mg/dL**	1.1 [0.4, 2.6]	0.2 [0, 0.3]	1.1 [0.7, 1.7]	4.5 [3.3, 6.7]	**<0.001**	F = 217.44	
**Median UA variability Delta mg/dL**	0.01 [−0.7, 1.5]	−0.94 [−1.0, −0.8]	0.01 [−0.4, 0.6]	3.40 [2.2, 5.5]	**<0.001**	F = 48.85	
**Trajectory increased (%)**	9962 (55%)	947 (58%)	1960 (60%)	955 (59%)	0.073		

Abbreviations: ACKD, advanced chronic kidney disease; AF, atrial fibrillation; BB, beta-blocker; CCB, calcium channel blocker; COPD, chronic obstructive pulmonary disease; CVA, cerebrovascular accident; DM, diabetes mellitus; eGFR, estimated glomerular filtration rate; HFpEF, heart failure with preserved ejection fraction; HTN, hypertension; IHD, ischemic heart disease; LVEDD, left ventricular end-diastolic diameter; LVEF, left ventricular ejection fraction; MRA, mineralocorticoid receptor antagonist; RAASi, renin–angiotensin–aldosterone system inhibitor; SGLT2i, sodium–glucose cotransporter-2 inhibitor; SPAP, systolic pulmonary artery pressure; TAPSE, tricuspid annular plane systolic excursion. Data are presented as median [interquartile range] or n (%), as appropriate. *p*-values were calculated using ANOVA for continuous variables and chi-square tests for categorical variables. Bold values indicate statistically significant differences (*p* < 0.05).

**Table 2:** Baseline characteristics by UA variability after PSM: Balanced comparison of clinical, laboratory, and echocardiographic parameters across Q1, Q2–Q3, and Q4 groups following PSM. Propensity score matching was performed using continuous LVEF values.

Variable	Low (Q1) (N = 2096)	Mid (Q2–Q3) (N = 4192)	High (Q4) (N = 2096)	*p*-Value
***General characteristics***				
**Age (Y)**	74 [64, 82]	74 [65, 82]	75 [66, 83]	**<0.001**
**Male (%)**	1247 (59%)	2516 (60%)	1242 (59%)	0.824
***Comorbidities***				
**ACKD (%)**	209 (10%)	650 (15%)	356 (17%)	**<0.001**
**HTN (%)**	1425 (68%)	3009 (72%)	1529 (73%)	**0.001**
**DM (%)**	1166 (56%)	2548 (61%)	1367 (65%)	**<0.001**
**AF (%)**	1049 (50%)	2295 (55%)	1251 (60%)	**<0.001**
**CVA (%)**	483 (23%)	1043 (25%)	530 (25%)	0.180
**COPD (%)**	266 (13%)	670 (16%)	391 (19%)	**<0.001**
**IHD (%)**	1065 (51%)	2244 (53%)	1169 (56%)	**0.005**
**Anemia (%)**	1143 (54%)	2652 (63%)	1492 (71%)	**<0.001**
***Medications***				
**RAASi (%)**	1276 (61%)	2575 (61%)	1291 (61%)	0.879
**BB (%)**	1484 (71%)	2977 (71)	1514 (72%)	0.521
**CCB (%)**	674 (32%)	1344 (32%)	637 (30%)	0.348
**Furosemide (%)**	1521 (73%)	3017 (72%)	1523 (73%)	0.803
**Thiazides (%)**	177 (9%)	389 (10%)	225 (11%)	**0.036**
**MRA (%)**	464 (22%)	956 (23%)	484 (23%)	0.746
**SGLT2i (%)**	141 (7%)	282 (7%)	139 (7%)	0.989
**UA-lowering medications (%)**	309 (6.5%)	323 (7%)	398 (8.5%)	**<0.001**
***Lab tests***				
**eGFR mL/min/1.73 m^2^**	70 [50, 89]	62 [43, 85]	54 [34, 76]	**<0.001**
***Echocardiographic parameters***				
**LVEF (%)**	50.0 [32.0, 60.0]	50.0 [33.0, 60.0]	50.0 [30.0, 60.0]	0.646
**HFpEF (%)**	628 (30%)	1714 (41%)	982 (47%)	**<0.001**
**SPAP (mmHg)**	38.0 [31.0, 48.0]	40.0 [32.0, 50.0]	43.0 [34.0, 54.0]	**<0.001**
**LVEDD (mm)**	4.90 [4.4, 5.5]	4.90 [4.4, 5.5]	4.96 [4.5, 5.5]	**0.003**
**TAPSE (cm)**	2.0 [1.9, 2.2]	2.0 [1.4, 2.5]	1.6 [1.2, 1.7]	0.300
***UA-related parameters***				
**Baseline First UA mg/dL**	6.14 [4.8, 7.7]	6.65 [5.3, 8.1]	7.90 [6.4, 9.6]	**0.001**
**Median UA variability mg/dL**	0.22 [0.1, 0.3]	1.15 [0.7, 1.7]	4.39 [3.3, 6.5]	**<0.001**
**Median UA variability Delta mg/dL**	−0.93 [−1.0, −0.8]	0.04 [−0.3, 0.6]	3.31 [2.2, 5.5]	**0.001**
**Trajectory increased (%)**	1142 (54%)	2343 (56%)	1162 (55%)	0.571

Abbreviations: ACKD, advanced chronic kidney disease; AF, atrial fibrillation; BB, beta-blocker; CCB, calcium channel blocker; COPD, chronic obstructive pulmonary disease; CVA, cerebrovascular accident; DM, diabetes mellitus; eGFR, estimated glomerular filtration rate; HFpEF, heart failure with preserved ejection fraction; HTN, hypertension; IHD, ischemic heart disease; LVEDD, left ventricular end-diastolic diameter; LVEF, left ventricular ejection fraction; MRA, mineralocorticoid receptor antagonist; RAASi, renin–angiotensin–aldosterone system inhibitor; SGLT2i, sodium–glucose cotransporter-2 inhibitor; SPAP, systolic pulmonary artery pressure; TAPSE, tricuspid annular plane systolic excursion. Data are presented as median [interquartile range] or n (%), as appropriate. *p*-values were calculated using ANOVA for continuous variables and chi-square tests for categorical variables. Bold values indicate statistically significant differences (*p* < 0.05).

**Table 3:** Univariate and multivariate Cox regression analyses of mortality predictors pre PSM: Univariate hazard ratios (HR, 95% CI) for mortality and multivariate models adjusted for major clinical covariates.

	Univariate Analysis				Multivariate Analysis			
Variable	HR	CI Lower	CI Upper	*p*-Value	HR	CI Lower	CI Upper	*p*-Value
**≤** **Q1 vs. Q2** **–** **Q3**	0.73	0.70	0.77	**<0.001**	0.79	0.75	0.83	**<0.001**
**≥** **Q4 vs. Q2** **–** **Q3**	1.83	1.75	1.91	**<0.001**	1.59	1.52	1.66	**<0.001**
**Age**	1.05	1.05	1.05	**<0.001**	1.05	1.04	1.05	**<0.001**
**EF < 40%**	1.09	1.05	1.14	**<0.001**	1.21	1.16	1.26	**<0.001**
**SPAP > 40**	1.64	1.57	1.71	**<0.001**	1.21	1.17	1.26	**<0.001**
**eGFR** **≤** **30**	2.08	1.97	2.20	**<0.001**	1.38	1.31	1.46	**<0.001**
**BMI > 30**	0.78	0.74	0.82	**<0.001**	0.82	0.78	0.86	**<0.001**
**AF**	1.16	1.12	1.21	**<0.001**	0.9	0.87	0.94	**<0.001**
**IHD**	1.01	0.97	1.04	0.680	0.91	0.88	0.95	**<0.001**
**HTN**	1.14	1.09	1.18	**<0.001**	0.86	0.83	0.9	**<0.001**
**CVA**	1.19	1.14	1.24	**<0.001**	1.1	1.06	1.15	**<0.001**
**COPD**	1.30	1.24	1.36	**<0.001**	1.21	1.16	1.27	**<0.001**
**GDMT**	1.02	0.97	1.08	0.469	1.04	1	1.09	0.06
**Anemia**	1.82	1.75	1.89	**<0.001**	1.45	1.4	1.51	**<0.001**
**DM**	0.96	0.93	1.00	**0.038**	0.96	0.92	0.99	**0.02**
**Baseline UA**	1.10	1.09	1.11	**<0.001**	1.1	1.08	1.12	**<0.001**

Abbreviations: AF, atrial fibrillation; CI, confidence interval; COPD, chronic obstructive pulmonary disease; CVA, cerebrovascular accident; DM, diabetes mellitus; EF, ejection fraction; eGFR, estimated glomerular filtration rate; HTN, hypertension; HR, hazard ratio; IHD, ischemic heart disease; SPAP, systolic pulmonary artery pressure. Bold values indicate statistically significant differences (*p* < 0.05).

**Table 4:** Univariate and multivariate Cox regression analyses of HF-related hospitalizations predictors pre PSM: Univariate and multivariate hazard ratios (HR, 95% CI) for mortality and multivariate models adjusted for major clinical covariates evaluating HF-related hospitalization.

	Univariate Analysis				Multivariate Analysis			
	HR	CI Lower	CI Upper	*p*-Value	HR	CI Lower	CI Upper	*p*-Value
≤Q1 vs. Q2–Q3	0.81	0.77	0.86	**<0.001**	0.85	0.80	0.90	**<0.001**
≥Q4 vs. Q2–Q3	1.26	1.19	1.34	**<0.001**	1.18	1.11	1.25	**<0.001**
Age	1.00	1.00	1.01	0.890	1.00	1.00	1.00	0.894
EF < 40%	1.42	1.34	1.49	**<0.001**	1.34	1.27	1.42	**<0.001**
SPAP > 40	1.27	1.20	1.33	**<0.001**	1.12	1.06	1.18	**<0.001**
eGFR < 30	1.27	1.18	1.36	**<0.001**	1.07	0.99	1.15	0.094
BMI > 30	0.91	0.86	0.97	0.003	0.94	0.89	1	0.05
AF	0.96	0.91	1.01	0.079	0.93	0.88	0.98	**0.004**
IHD	1.03	0.98	1.08	0.268	1	0.95	1.05	0.949
HTN	0.88	0.83	0.93	**<0.001**	0.87	0.82	0.93	**<0.001**
CVA	0.90	0.85	0.95	**<0.001**	0.91	0.86	0.97	**0.001**
COPD	0.95	0.89	1.01	0.084	0.95	0.89	1.01	0.087
GDMT	0.98	0.91	1.05	0.553	0.98	0.93	1.04	0.577
Anemia	1.22	1.16	1.28	**<0.001**	1.16	1.1	1.23	**<0.001**
DM	0.97	0.92	1.02	0.265	0.99	0.94	1.04	0.719
Baseline UA	1.06	1.05	1.07	**<0.001**	1.1	1.07	1.13	**<0.001**

Abbreviations: AF, atrial fibrillation; CI, confidence interval; COPD, chronic obstructive pulmonary disease; CVA, cerebrovascular accident; DM, diabetes mellitus; EF, ejection fraction; eGFR, estimated glomerular filtration rate; HTN, hypertension; HR, hazard ratio; IHD, ischemic heart disease; SPAP, systolic pulmonary artery pressure. Bold values indicate statistically significant differences (*p* < 0.05).

## Data Availability

The data that support the findings of this study are derived from the SHEBAHEART registry and are not publicly available due to privacy and institutional governance restrictions. De-identified data may be made available upon reasonable request and subject to institutional approvals and a data-sharing agreement.

## Supplementary Materials

The following supporting information can be downloaded at: https://www.mdpi.com/article/10.3390/jcm15062330/s1, Table S1. Unadjusted associations between uric acid variability and mortality. Table S2. Multivariable Cox Model of Mortality in the Overall HF Cohort. Table S3. Unadjusted PSM-Weighted Associations Between Uric Acid Variability and Mortality. Table S4. Multivariable PSM-Adjusted SDM Values. Table S5. Multivariable PSM-Adjusted Cox Model of Mortality in the Overall Cohort. Table S6. Unadjusted Associations Between Uric Acid Variability and HF-Related Hospitalization. Table S7. Multivariable Cox Model of HF-Related Hospitalization in the Overall HF Cohort. Table S8. Unadjusted PSM-Weighted Associations Between Uric Acid Variability and HF-Related Hospitalization. Table S9. Multivariable PSM-Adjusted Cox Model of HF-Related Hospitalization in the Overall Cohort. Table S10. Multivariable Cox Model of Mortality in HFrEF Patients. Table S11. Multivariable Cox Model of Mortality in HFpEF Patients. Table S12. Multivariable Cox Model of Mortality in Patients Without Oncological Disease. Table S13. Multivariable Cox Model of Mortality in Patients Without COPD. Table S14. Multivariable Cox Model of Mortality in Male Patients. Table S15. Multivariable Cox Model of Mortality in Female Patients. Table S16. Multivariable Cox Model of Mortality in Octogenarians (≥80 years). Table S17. Multivariable Cox Model of Mortality in Non-Octogenarians (<80 years). Table S18. Multivariable Cox Model of Mortality in Patients Without CKD. Table S19. Multivariable Cox Model of Mortality in Patients With CKD. Table S20. Multivariable Cox Model of Mortality in Patients Not Receiving Furosemide. Table S21. Multivariable Cox Model of Mortality in Patients Receiving Furosemide. Table S22. Multivariable Cox Model of Mortality in Patients Receiving UA Lowering Drugs. Table S23. Adjusted Cox Model Adjusted for TAPSE. Table S24. Unadjusted Associations Between Uric Acid Trajectories and Outcomes Across Variability Strata. Table S25. Adjusted Cox Model of UA Trajectories & Outcomes—Low Variability (≤Q1). Table S26. Adjusted Cox Model of UA Trajectories & Outcomes—Moderate Variability (Q2–Q3). Table S27. Adjusted Cox Model of UA Trajectories & Outcomes—High Variability (≥Q4). Table S28. Multivariable PSM-Adjusted Cox Model of Mortality—HFrEF. Table S29. Multivariable PSM-Adjusted Cox Model of Mortality—HFpEF. Table S30. Multivariable PSM-Adjusted Cox Model of Mortality—Without Oncological Disease. Table S31. Multivariable PSM-Adjusted Cox Model of Mortality—Without COPD. Table S32. Multivariable PSM-Adjusted Cox Model of Mortality—Male. Table S33. Multivariable PSM-Adjusted Cox Model of Mortality—Female. Table S34. Multivariable PSM-Adjusted Cox Model of Mortality—Octogenarians (≥80 years). Table S35. Multivariable PSM-Adjusted Cox Model of Mortality—<80 Years. Table S36. Multivariable PSM-Adjusted Cox Model of Mortality—With CKD. Table S37. Multivariable PSM-Adjusted Cox Model of Mortality—Without CKD. Table S38. Multivariable PSM-Adjusted Cox Model of Mortality—Receiving Furosemide. Table S39. Multivariable PSM-Adjusted Cox Model of Mortality—Not Receiving Furosemide. Table S40. Multivariable PSM-Adjusted Cox Model of Mortality—Receiving Allopurinol. Table S41. Multivariable Cox Model of HF-Related Hospitalization—HfrEF. Table S42. Multivariable Cox Model of HF-Related Hospitalization—HfpEF. Table S43. Multivariable Cox Model of HF-Related Hospitalization—Without Oncological Disease. Table S44. Multivariable Cox Model of HF-Related Hospitalization—Without COPD. Table S45. Multivariable Cox Model of HF-Related Hospitalization—Male. Table S46. Multivariable Cox Model of HF-Related Hospitalization—Female. Table S47. Multivariable Cox Model of HF-Related Hospitalization—Octogenarians (≥80 years). Table S48. Multivariable Cox Model of HF-Related Hospitalization—Non-Octogenarians (<80 years). Table S49. Multivariable Cox Model of HF-Related Hospitalization—Without CKD. Table S50. Multivariable Cox Model of HF-Related Hospitalization—With CKD. Table S51. Multivariable Cox Model of HF-Related Hospitalization—Receiving Furosemide. Table S52. Multivariable Cox Model of HF-Related Hospitalization—Not Receiving Furosemide. Table S53. Multivariable Cox Model of HF-Related Hospitalization—Receiving UA Lowering Drugs. Table S54. Adjusted Cox Model of HF-related Hospitalization Adjusted for TAPSE. Table S55. Unadjusted Associations Between UA Trajectories and HF-Related Hospitalization Across Variability Strata. Table S56. Adjusted Cox Model of HF-Related Hospitalization—Low UA Variability (≤Q1). Table S57. Adjusted Cox Model of HF-Related Hospitalization—Moderate UA Variability (Q2–Q3). Table S58. Adjusted Cox Model of HF-Related Hospitalization—High UA Variability (≥Q4). Table S59. Multivariable PSM-Adjusted Cox Model of HF-Related Hospitalization—HfrEF. Table S60. Multivariable PSM-Adjusted Cox Model of HF-Related Hospitalization—HfpEF. Table S61. Multivariable PSM-Adjusted Cox Model of HF-Related Hospitalization—Without Oncological Disease. Table S62. Multivariable PSM-Adjusted Cox Model of HF-Related Hospitalization—Without COPD. Table S63. Multivariable PSM-Adjusted Cox Model of HF-Related Hospitalization—Male. Table S64. Multivariable PSM-Adjusted Cox Model of HF-Related Hospitalization—Female. Table S65. Multivariable PSM-Adjusted Cox Model of HF-Related Hospitalization—Octogenarians (≥80 years). Table S66. Multivariable PSM-Adjusted Cox Model of HF-Related Hospitalization—<80 Years. Table S67. Multivariable PSM-Adjusted Cox Model of HF-Related Hospitalization—Without CKD. Table S68. Multivariable PSM-Adjusted Cox Model of HF-Related Hospitalization—With CKD. Table S69. Multivariable PSM-Adjusted Cox Model of HF-Related Hospitalization—Not Receiving Furosemide. Table S70. Multivariable PSM-Adjusted Cox Model of HF-Related Hospitalization—Receiving Furosemide. Table S71. Multivariable PSM-Adjusted Cox Model of HF-Related Hospitalization—Receiving Allopurinol. Figure S1. Study design. Figure S2. Rates of heart failure hospitalization. Figure S3. Survival by UA variability and heart failure subtype. Figure S4. Survival by UA variability and furosemide use.

## Abbreviations

HF	Heart failure
UA	Uric acid
EF	Ejection fraction
HFrEF	Heart failure with reduced ejection fraction
HFpEF	Heart failure with preserved ejection fraction
ACKD	Advanced chronic kidney disease
eGFR	Estimated glomerular filtration rate
GDMT	Guideline-directed medical therapy

## References

[1] Savarese G., Lund L.H. (2017). Global Public Health Burden of Heart Failure. Card. Fail. Rev..

[2] Grossman C., Grossman E., Goldbourt U. (2019). Uric acid variability at midlife as an independent predictor of coronary heart disease and all-cause mortality. PLoS ONE.

[3] Tian X., Chen S., Zhang Y., Zhang X., Xu Q., Wang P., Wu S., Luo Y., Wang A. (2023). Serum uric acid variation and the risk of cardiovascular disease: A prospective cohort study. Eur. J. Intern. Med..

[4] Gibbs J., Gaskin E., Ji C., Miller M.A., Cappuccio F.P. (2021). The effect of plant-based dietary patterns on blood pressure: A systematic review and meta-analysis of controlled intervention trials. J. Hypertens..

[5] Rahimi-Sakak F., Maroofi M., Rahmani J., Bellissimo N., Hekmatdoost A. (2019). Serum uric acid and risk of cardiovascular mortality: A systematic review and dose-response meta-analysis of cohort studies of over a million participants. BMC Cardiovasc. Disord..

[6] Miao L., Guo M., Pan D., Chen P., Chen Z., Gao J., Yu Y., Shi D., Du J. (2021). Serum Uric Acid and Risk of Chronic Heart Failure: A Systematic Review and Meta-Analysis. Front. Med..

[7] Selvaraj S., Claggett B.L., Pfeffer M.A., Desai A.S., Mc Causland F.R., McGrath M.M., Anand I.S., van Veldhuisen D.J., Kober L., Janssens S. (2020). Serum uric acid, influence of sacubitril–valsartan, and cardiovascular outcomes in heart failure with preserved ejection fraction: PARAGON-HF. Eur. J. Heart Fail..

[8] Stevens S.L., Wood S., Koshiaris C., Law K., Glasziou P., Stevens R.J., McManus R.J. (2016). Blood pressure variability and cardiovascular disease: Systematic review and meta-analysis. BMJ.

[9] Hirakawa Y., Arima H., Zoungas S., Ninomiya T., Cooper M., Hamet P., Mancia G., Poulter N., Harrap S., Woodward M. (2014). Impact of visit-to-visit glycemic variability on the risks of macrovascular and micro-vascular events in type 2 diabetes. Diabetes Care.

[10] Bangalore S., Breazna A., DeMicco D.A., Wun C.-C., Messerli F.H. (2015). Visit-to-visit low-density lipoprotein cholesterol variability and risk of cardiovascular outcomes. JACC.

[11] Tian X., Wang A., Zuo Y., Chen S., Zhang L., Wu S., Luo Y. (2021). Visit-to-visit variability of serum uric acid measurements and the risk of all-cause mortality in the general population. Arthritis Res Ther..

[12] Wu W.C., Lai Y.W., Chou Y.C., Liao Y.C., You S.L., Bai C.H., Sun C.A. (2020). Serum Uric Acid Level as a Harbinger of Type 2 Diabetes: A Prospective Observation in Taiwan. Int. J. Environ. Res. Public Health.

[13] Kim H.K., Lee M., Lee Y.-H., Lee B.-W., Cha B.-S., Kang E.S. (2021). Uric acid variability as a predictive marker of newly developed cardiovascular events in type 2 diabetes. Front. Cardiovasc. Med..

[14] Qin S., Xiang M., Gao L., Cheng X., Zhang D. (2024). Uric acid is a biomarker for heart failure, but not therapeutic target: Result from a comprehensive meta-analysis. ESC Heart Fail..

[15] Yan W., Tang H.-Y., Yang Y.-Q., He K.-L. (2023). Serum uric acid and outcome in hospitalized elderly patients with chronic heart failure through the whole spectrum of ejection fraction phenotypes. BMC Cardiovasc. Disord..

[16] Tamariz L., Harzand A., Palacio A., Verma S., Jones J., Hare J. (2011). Uric acid as a predictor of all-cause mortality in heart failure: A meta-analysis. Congest. Heart Fail..

[17] Wang M., Wang C., Zhao M., Li Y., Yao S., Wu S., Xue H. (2021). Uric Acid Variability and All-Cause Mortality: A Prospective Cohort Study in Northern China. J. Nutr. Health Aging.

[18] Copeland V., Fishman B., Milwidsky A., Makmal N., Loutati R., Elimeleh S., Hochstein D., Fardman A., Wasserstrum Y., Segev A. (2025). Improved Survival in Octogenarians with Heart Failure. JACC Adv..

[19] McDonagh T.A., Metra M., Adamo M., Gardner R.S., Baumbach A., Böhm M., Burri H., Butler J., Čelutkienė J., Chioncel O. (2023). 2023 focused update of the 2021 ESC guidelines for the diagnosis and treatment of acute and chronic heart failure. Eur. Heart J..

[20] Hicks K.A., Mahaffey K.W., Mehran R., Nissen S.E., Wiviott S.D., Dunn B., Solomon S.D., Marler J.R., Teerlink J.R., Farb A. (2018). 2017 Cardiovascular and Stroke Endpoint Definitions for Clinical Trials. Circulation.

[21] Wilkinson C., Bhatty A., Batra G., Aktaa S., Smith A.B., Dwight J., Rucinski M., Chappell S., Alfredsson J., Erlinge D. (2025). Definitions of clinical study outcome measures for cardiovascular diseases: The European Unified Registries for Heart Care Evaluation and Randomized Trials (EuroHeart). Eur. Heart J..

[22] Song Z., Deng D., Wu H. (2024). Association of serum uric acid to all-cause and cardiovascular mortality in patients with cardiovascular disease. Sci. Rep..

[23] Lim S.S., Yang Y.-L., Chen S.-C., Wu C.-H., Huang S.-S., Chan W.L., Lin S.-J., Chen J.-W., Chou C.-Y., Pan J.-P. (2020). Association of variability in uric acid and future clinical outcomes of patient with coronary artery disease undergoing percutaneous coronary intervention. Atherosclerosis.

[24] Moshkovits Y., Tiosano S., Kaplan A., Kalstein M., Bayshtok G., Kivity S., Segev S., Grossman E., Segev A., Maor E. (2023). Serum uric acid significantly improves the accuracy of cardiovascular risk score models. Eur. J. Prev. Cardiol..

[25] Grossman C., Shemesh J., Koren-Morag N., Bornstein G., Ben-Zvi I., Grossman E. (2014). Serum Uric Acid Is Associated with Coronary Artery Calcification. J. Clin. Hypertens..

[26] Weinstein S., Maor E., Bleier J., Kaplan A., Hod T., Leibowitz A., Grossman E., Shlomai G. (2024). Non-Interventional Weight Changes Are Associated with Alterations in Serum Uric Acid Levels. J. Clin. Med..

[27] Ambrosio G., Leiro M.G.C., Lund L.H., Coiro S., Cardona A., Filippatos G., Ferrari R., Piepoli M.F., Coats A.J.S., Anker S.D. (2021). Serum uric acid and outcomes in patients with chronic heart failure through the whole spectrum of ejection fraction phenotypes: Analysis of the ESC-EORP Heart Failure Long-Term (HF LT) Registry. Eur. J. Intern. Med..

[28] Li L., Chang Y., Li F., Yin Y. (2024). Relationship between serum uric acid levels and uric acid lowering therapy with the prognosis of patients with heart failure with preserved ejection fraction: A meta-analysis. Front. Cardiovasc. Med..

[29] Kaufman M., Guglin M. (2013). Uric acid in heart failure: A biomarker or therapeutic target?. Heart Fail. Rev..

[30] Huang H., Huang B., Li Y., Huang Y., Li J., Yao H., Jing X., Chen J., Wang J. (2014). Uric acid and risk of heart failure: A systematic review and meta-analysis. Eur. J. Heart Fail..

[31] Lu S.Y., Hsu H.Y., Hsieh Y.W., Lu C.R., Huang H.Y., Chang S.S. (2024). Prescribing cascades of antigout medications from thiazide diuretics in gout-naïve hypertensive adults receiving first-line pharmacological management. Sci. Rep..

[32] Testani J.M., Chen J., McCauley B.D., Kimmel S.E., Shannon R.P. (2010). Potential effects of aggressive decongestion during the treatment of decompensated heart failure on renal function and survival. Circulation.

[33] Şahin S.I., Öztürk E.Ç., Öztürk H., Aydın B.Ç., Cezairli F.P.Z., Hoca E., Ataoğlu H.E. (2025). Low Serum Uric Acid as an Independent Predictor of Mortality and Poor Prognosis: A Retrospective Cohort Study. J. Clin. Med..

[34] Muk B., Bánfi-Bacsárdi F., Vámos M., Pilecky D., Majoros Z., Török G.M., Vágány D., Polgár B., Solymossi B., Borsányi T.D. (2024). The Impact of Specialised Heart Failure Outpatient Care on the Long-Term Application of Guideline-Directed Medical Therapy and on Prognosis in Heart Failure with Reduced Ejection Fraction. Diagnostics.

[35] Behar D.M., Yunusbayev B., Metspalu M., Metspalu E., Rosset S., Parik J., Rootsi S., Chaubey G., Kutuev I., Yudkovsky G. (2010). The genome-wide structure of the Jewish people. Nature.

[36] Kasiak P.S., Buchalska B., Kowalczyk W., Wyszomirski K., Krzowski B., Grabowski M., Balsam P. (2022). The Path of a Cardiac Patient—From the First Symptoms to Diagnosis to Treatment: Experiences from the Tertiary Care Center in Poland. J. Clin. Med..

