# Supplementary material for: Uric Acid Variability Is Associated with Poor Prognosis in Heart Failure
Source: J Clin Med. 2026 Mar 18;15(6):2330. doi: 10.3390/jcm15062330 (PMC13026695; doi:10.3390/jcm15062330)
Supplement: Supplementary file 1 [file jcm-15-02330-s001.zip › jcm-4179270-supplementary.pdf]

Core models

Table S1. *Unadjusted Associations Between Uric Acid Variability and Mortality - Page 6*

Table S2. *Multivariable Cox Model of Mortality in the Overall HF Cohort- Page 6*

Table S3. *Unadjusted PSM-Weighted Associations Between Uric Acid Variability and Mortality - Page 6*

Table S4. *Multivariable PSM-Adjusted SDM Values - Page 7*

Table S5. *Multivariable PSM-Adjusted Cox Model of Mortality in the Overall Cohort - Page 7*

Table S6. *Unadjusted Associations Between Uric Acid Variability and HF-Related Hospitalization - Page 8*

Table S7. *Multivariable Cox Model of HF-Related Hospitalization in the Overall HF Cohort - Page 8*

Table S8. *Unadjusted PSM-Weighted Associations Between Uric Acid Variability and HF-Related Hospitalization - Page 8*

Table S9. *Multivariable PSM-Adjusted Cox Model of HF-Related Hospitalization in the Overall Cohort - Page 9*

Mortality subgroup and sensitivity analysis

Table S10. *Multivariable Cox Model of Mortality in HFrEF Patients- Page 10*

Table S11. *Multivariable Cox Model of Mortality in HFpEF Patients - Page 10*

Table S12. *Multivariable Cox Model of Mortality in Patients Without Oncological Disease - Page 11*

Table S13. *Multivariable Cox Model of Mortality in Patients Without COPD - Page 11*

Table S14. *Multivariable Cox Model of Mortality in Male Patients - Page 12*

Table S15. *Multivariable Cox Model of Mortality in Female Patients - Page 13*

Table S16. *Multivariable Cox Model of Mortality in Octogenarians (≥80 years) - Page 13*

Table S17. *Multivariable Cox Model of Mortality in Non-Octogenarians (<80 years) - Page 14*

Table S18. *Multivariable Cox Model of Mortality in Patients Without CKD - Page 14*

Table S19. *Multivariable Cox Model of Mortality in Patients With CKD - Page 14*

Table S20. *Multivariable Cox Model of Mortality in Patients Not Receiving Furosemide - Page 15*

Table S21. *Multivariable Cox Model of Mortality in Patients Receiving Furosemide - Page 16*

Table S22. *Multivariable Cox Model of Mortality in Patients Receiving UA Lowering Drugs - Page 17*

Table S23. *Adjusted Cox Model Adjusted for TAPSE - Page 17*

Table S24. *Unadjusted Associations Between Uric Acid Trajectories and Outcomes Across Variability Strata - Page 18*

Table S25. *Adjusted Cox Model of UA Trajectories & Outcomes - Low Variability (≤Q1) - Page 18*

Table S26. *Adjusted Cox Model of UA Trajectories & Outcomes - Moderate Variability (Q2–Q3) - Page 19*

Table S27. *Adjusted Cox Model of UA Trajectories & Outcomes - High Variability (≥Q4) - Page 19*

PSM-weighted mortality

Table S28. *Multivariable PSM-Adjusted Cox Model of Mortality - HFrEF - Page 21*

Table S29. *Multivariable PSM-Adjusted Cox Model of Mortality - HFpEF - Page 21*

Table S30. *Multivariable PSM-Adjusted Cox Model of Mortality - Without Oncological Disease - Page 22*

Table S31. *Multivariable PSM-Adjusted Cox Model of Mortality - Without COPD - Page 22*

Table S32. *Multivariable PSM-Adjusted Cox Model of Mortality - Male - Page 23*

Table S33. *Multivariable PSM-Adjusted Cox Model of Mortality - Female - Page 23*

Table S34. *Multivariable PSM-Adjusted Cox Model of Mortality - Octogenarians (≥80 years) - Page 24*

Table S35. *Multivariable PSM-Adjusted Cox Model of Mortality - <80 Years - Page 25*

Table S36. *Multivariable PSM-Adjusted Cox Model of Mortality - With CKD - Page 25*

Table S37. *Multivariable PSM-Adjusted Cox Model of Mortality - Without CKD - Page 26*

Table S38. *Multivariable PSM-Adjusted Cox Model of Mortality - Receiving Furosemide - Page 26*

Table S39. *Multivariable PSM-Adjusted Cox Model of Mortality - Not Receiving Furosemide - Page 27*

Table S40. *Multivariable PSM-Adjusted Cox Model of Mortality - Receiving Allopurinol - Page 28*

HF related hospitalization subgroup and sensitivity analysis

Table S41. *Multivariable Cox Model of HF-Related Hospitalization - HFrEF - Page 29*

Table S42. *Multivariable Cox Model of HF-Related Hospitalization - HFpEF - Page 29*

Table S43. *Multivariable Cox Model of HF-Related Hospitalization - Without Oncological Disease - Page 30*

Table S44. *Multivariable Cox Model of HF-Related Hospitalization - Without COPD - Page 30*

Table S45. *Multivariable Cox Model of HF-Related Hospitalization - Male - Page 31*

Table S46. *Multivariable Cox Model of HF-Related Hospitalization - Female - Page 32*

Table S47. *Multivariable Cox Model of HF-Related Hospitalization - Octogenarians (≥80 years) - Page 32*

Table S48. *Multivariable Cox Model of HF-Related Hospitalization - Non-Octogenarians (<80 years) - Page 33*

Table S49. *Multivariable Cox Model of HF-Related Hospitalization - Without CKD - Page 33*

Table S50. *Multivariable Cox Model of HF-Related Hospitalization - With CKD - Page 34*

Table S51. *Multivariable Cox Model of HF-Related Hospitalization - Receiving Furosemide- Page 34*

Table S52. *Multivariable Cox Model of HF-Related Hospitalization - Not Receiving Furosemide - Page 35*

Table S53. *Multivariable Cox Model of HF-Related Hospitalization - Receiving UA Lowering Drugs- Page 36*

Table S54. *Adjusted Cox Model of HF-related Hospitalization Adjusted for TAPSE - Page 19*

Table S55. *Unadjusted Associations Between UA Trajectories and HF-Related Hospitalization Across Variability Strata - Page 37*

Table S56. *Adjusted Cox Model of HF-Related Hospitalization - Low UA Variability (≤Q1) - Page 37*

Table S57. *Adjusted Cox Model of HF-Related Hospitalization - Moderate UA Variability (Q2–Q3) - Page 38*

Table S58. *Adjusted Cox Model of HF-Related Hospitalization - High UA Variability (≥Q4) - Page 38*

PSM-weighted HF related hospitalization

Table S59. *Multivariable PSM-Adjusted Cox Model of HF-Related Hospitalization - HFrEF - Page 40*

Table S60. *Multivariable PSM-Adjusted Cox Model of HF-Related Hospitalization - HFpEF - Page 40*

Table S61. *Multivariable PSM-Adjusted Cox Model of HF-Related Hospitalization - Without Oncological Disease - Page 41*

Table S62. *Multivariable PSM-Adjusted Cox Model of HF-Related Hospitalization - Without COPD - Page 41*

Table S63. *Multivariable PSM-Adjusted Cox Model of HF-Related Hospitalization - Male - Page 42*

Table S64. *Multivariable PSM-Adjusted Cox Model of HF-Related Hospitalization - Female - Page 43*

Table S65. *Multivariable PSM-Adjusted Cox Model of HF-Related Hospitalization - Octogenarians (≥80 years) - Page 43*

Table S66. *Multivariable PSM-Adjusted Cox Model of HF-Related Hospitalization - <80 Years - Page 44*

Table S67. *Multivariable PSM-Adjusted Cox Model of HF-Related Hospitalization - Without CKD - Page 44*

Table S68. *Multivariable PSM-Adjusted Cox Model of HF-Related Hospitalization - With CKD - Page 45*

Table S69. *Multivariable PSM-Adjusted Cox Model of HF-Related Hospitalization - Not Receiving Furosemide - Page 46*

Table S70. *Multivariable PSM-Adjusted Cox Model of HF-Related Hospitalization - Receiving Furosemide - Page 46*

Table S71. *Multivariable PSM-Adjusted Cox Model of HF-Related Hospitalization - Receiving Allopurinol - Page 47*

Figures

Figure S1. *Study design* ***-*** *Page 48*

Figure S2. *Rates of heart failure hospitalization - Page 48*

Figure S3. *Survival by UA variability and heart failure subtype - Page 49*

Figure S4. *Survival by UA variability and furosemide use - Page 50*

Core models

Contrast	HR and CI	p-value
≤Q1 vs Q2-Q3	0.73 (0.69-0.76)	**<0.001**
≥Q4 vs Q2-Q3	1.82 (1.75-1.90)	**<0.001**

Table S1. Unadjusted Associations Between Uric Acid Variability and Mortality: *Unadjusted Cox models showing the hazard ratios (HRs) for lowest (≤Q1) and highest (≥Q4) uric acid variability quartiles compared with the reference (Q2-Q3).*

Variable	HR	CI Lower	CI Upper	p-value
≤Q1 vs Q2-Q3	0.79	0.75	0.83	**<0.001**
≥Q4 vs Q2-Q3	1.59	1.52	1.66	**<0.001**
Age	1.05	1.04	1.05	**<0.001**
EF < 40%	1.21	1.16	1.26	**<0.001**
SPAP > 40	1.21	1.17	1.26	**<0.001**
GFR < 30	1.38	1.31	1.46	**<0.001**
BMI > 30	0.82	0.78	0.86	**<0.001**
AF	0.90	0.87	0.94	**<0.001**
IHD	0.91	0.88	0.95	**<0.001**
HTN	0.86	0.83	0.90	**<0.001**
CVA	1.10	1.06	1.15	**<0.001**
COPD	1.21	1.16	1.27	**<0.001**
GDMT	1.04	1.00	1.09	0.060
Anemia	1.45	1.40	1.51	**<0.001**
DM	0.96	0.92	0.99	**0.020**
Baseline UA	1.10	1.08	1.12	**<0.001**

Table S2. Multivariable Cox Model of Mortality in the Overall HF Cohort: *Fully adjusted Cox regression including uric acid variability, demographics, comorbidities, and clinical covariates.*

Contrast	HR and CI	p-value
≤Q1 vs Q2-Q3	1.01 (0.96-1.06)	0.570
≥Q4 vs Q2-Q3	1.45 (1.38-1.51)	**<0.001**

Table S3. Unadjusted PSM-Weighted Associations Between Uric Acid Variability and Mortality: *Propensity score-matched (PSM) unadjusted Cox models comparing Q1 and Q4 with the reference (Q2-Q3). Hazard ratios with 95% confidence intervals (HR, CI) and p-values are shown.*

Variable	SMD Before	SMD After
**ACE**	0.020	-0.012

**AF**	-0.135	-0.018
**Age**	-0.096	-0.011
**Anemia**	-0.004	-0.006
**BB**	0.010	-0.002
**BMI**	-0.032	0.021
**COPD**	-0.127	0.013
**CVA**	-0.112	-0.006
**distance**	0.404	0.028
**DM**	-0.150	-0.008
**Echo LVEF**	-0.022	0.004
**Echo SPAP**	-0.138	-0.008
**Entresto**	0.021	0.021
**GFR**	0.192	0.013
**HTN**	-0.139	-0.019
**IHD**	-0.112	0.015
**Missing ACE**	-0.012	0.000
**Missing BB**	-0.012	0.000
**Missing BMI**	0.061	-0.007
**Missing Echo LVEF**	0.173	0.044
**Missing Echo SPAP**	0.126	0.032
**Missing Entresto**	-0.012	0.000
**Missing GFR**	0.140	-0.011
**Missing MRA**	-0.012	0.000
**Missing SGLT2**	-0.012	0.000
**MRA**	-0.015	-0.002
**SGLT2**	0.038	0.027
**UA Baseline Value**	-0.217	0.005

Table S4: Balance of Baseline Covariates Before and After Propensity Score Matching- Standardized mean differences (SMDs) for baseline characteristics before and after propensity score matching.

Variable	HR	CI Lower	CI Upper	p-value
≤Q1 vs Q2-Q3	0.86	0.81	0.91	**<0.001**
≥Q4 vs Q2-Q3	1.51	1.44	1.58	**<0.001**
Age	1.05	1.04	1.05	**<0.001**

EF < 40%	1.17	1.11	1.24	**<0.001**
SPAP > 40	1.28	1.21	1.35	**<0.001**
GFR < 30	1.54	1.43	1.65	**<0.001**
BMI > 30	0.81	0.76	0.86	**<0.001**
AF	0.90	0.85	0.94	**<0.001**
IHD	0.98	0.93	1.04	0.500
HTN	0.89	0.83	0.95	**<0.001**
CVA	1.15	1.08	1.22	**<0.001**
COPD	1.31	1.23	1.41	**<0.001**
GDMT	1.09	1.02	1.16	**0.007**
Anemia	1.52	1.44	1.61	**<0.001**
DM	1.03	0.98	1.09	0.254
Baseline UA	1.23	1.19	1.26	**<0.001**

Table S5. Multivariable PSM-Adjusted Cox Model of Mortality in the Overall Cohort: *Fully adjusted PSM Cox regression including treatment contrasts (Q1 vs Q2-Q3; Q4 vs Q2-Q3) and clinical covariates.*

**Contrast**	**HR and CI**	**p-value**
≤Q1 vs Q2-Q3	0.81 (0.76-0.86)	**<0.001**
≥Q4 vs Q2-Q3	1.26 (1.19-1.34)	**<0.001**

Table S6. Unadjusted Associations Between Uric Acid Variability and HF-Related Hospitalization: *Unadjusted Cox models showing hazard ratios (HRs) for lowest (≤Q1) and highest (≥Q4) uric acid variability quartiles compared with the reference (Q2-Q3).*

**Variable**	**HR**	**CI Lower**	**CI Upper**	**p-value**
≤Q1 vs Q2-Q3	0.85	0.80	0.90	**<0.001**
≥Q4 vs Q2-Q3	1.18	1.11	1.25	**<0.001**
Age	1.00	1.00	1.00	**0.022**
EF < 40%	1.34	1.27	1.42	**<0.001**
SPAP > 40	1.12	1.06	1.18	**<0.001**
GFR < 30	1.07	0.99	1.15	0.094
BMI > 30	0.94	0.89	1.00	0.050
AF	0.93	0.88	0.98	**0.004**

IHD	1.00	0.95	1.05	0.949
HTN	0.87	0.82	0.93	**<0.001**
CVA	0.91	0.86	0.97	**0.001**
COPD	0.95	0.89	1.01	0.087
GDMT	0.98	0.93	1.04	0.577
Anemia	1.16	1.10	1.23	**<0.001**
DM	0.99	0.94	1.04	0.719
Baseline UA	1.10	1.07	1.13	**<0.001**

Table S7. Multivariable Cox Model of HF-Related Hospitalization in the Overall HF Cohort: *Fully adjusted Cox regression including uric acid variability, demographics, comorbidities, and clinical covariates.*

Contrast	HR and CI	p-value
Q1 vs Q2-Q3	0.84 (0.78-0.90)	**<0.001**
Q4 vs Q2-Q3	1.21 (1.13-1.30)	**<0.001**

Table S8. Unadjusted PSM-Weighted Associations Between Uric Acid Variability and HF-Related Hospitalization: *Propensity score-matched (PSM) unadjusted Cox models comparing Q1 and Q4 with the reference (Q2-Q3); HRs with 95% CIs and p-values shown.*

**Variable**	**HR**	**CI Lower**	**CI Upper**	**p-value**
≤Q1 vs Q2-Q3	0.86	0.80	0.93	**<0.001**
≥Q4 vs Q2-Q3	1.19	1.11	1.28	**<0.001**
Age	1.00	1.00	1.01	0.066
EF < 40%	1.34	1.22	1.47	**<0.001**
SPAP > 40	1.12	1.04	1.21	**0.003**
GFR < 30	1.12	0.98	1.27	0.085
BMI > 30	0.90	0.83	0.97	**0.009**
AF	0.89	0.83	0.96	**0.002**
IHD	0.98	0.91	1.05	0.539
HTN	0.87	0.80	0.95	**0.002**
CVA	0.88	0.81	0.95	**0.001**
COPD	0.93	0.85	1.01	0.087
GDMT	1.01	0.92	1.09	0.906
Anemia	1.15	1.07	1.24	**<0.001**
DM	0.97	0.90	1.04	0.355
Baseline UA	1.11	1.06	1.16	**<0.001**

Table S9. Multivariable PSM-Adjusted Cox Model of HF-Related Hospitalization in the Overall Cohort: *Fully adjusted PSM Cox regression including treatment contrasts (Q1 vs Q2-Q3; Q4 vs Q2-Q3) and covariates*

Mortality subgroup and sensitivity models

Variable	HR	CI Lower	CI Upper	p-value
≤Q1 vs Q2-Q3	0.79	0.75	0.83	**<0.001**
≥Q4 vs Q2-Q3	1.59	1.52	1.66	**<0.001**
Age	1.05	1.04	1.05	**<0.001**
SPAP > 40	1.21	1.17	1.26	**<0.001**
GFR < 30	1.38	1.31	1.46	**<0.001**
BMI > 30	0.82	0.78	0.86	**<0.001**
AF	0.90	0.87	0.94	**<0.001**
IHD	0.91	0.88	0.95	**<0.001**
HTN	0.86	0.83	0.90	**<0.001**
CVA	1.10	1.06	1.15	**<0.001**
COPD	1.21	1.16	1.27	**<0.001**
GDMT	1.04	1.00	1.09	0.065
Anemia	1.45	1.40	1.51	**<0.001**
DM	0.96	0.92	0.99	**0.025**
Baseline UA	1.10	1.08	1.12	**<0.001**

Table S10. Multivariable Cox Model of Mortality in HFrEF Patients: *Adjusted associations between uric acid variability and mortality among patients with reduced ejection fraction.*

Variable	HR	CI Lower	CI Upper	p-value
≤Q1 vs Q2-Q3	0.81	0.76	0.87	**<0.001**
≥Q4 vs Q2-Q3	1.69	1.59	1.80	**<0.001**
Age	1.05	1.04	1.05	**<0.001**
SPAP > 40	1.16	1.10	1.23	**<0.001**
GFR < 30	1.38	1.27	1.49	**<0.001**
BMI > 30	0.83	0.78	0.89	**<0.001**
AF	0.88	0.83	0.93	**<0.001**
IHD	0.83	0.79	0.88	**<0.001**
HTN	0.83	0.78	0.89	**<0.001**
CVA	1.09	1.02	1.15	**0.006**
COPD	1.24	1.16	1.32	**<0.001**
GDMT	1.00	0.94	1.07	0.890
Anemia	1.41	1.33	1.49	**<0.001**
DM	0.99	0.94	1.05	0.725
Baseline UA	1.06	1.03	1.09	**<0.001**

Table S11. Multivariable Cox Model of Mortality in HFpEF Patients: *Adjusted associations between uric acid variability and mortality among patients with preserved ejection fraction.*

Variable	HR	CI Lower	CI Upper	p-value
≤Q1 vs Q2-Q3	0.79	0.75	0.84	**<0.001**
≥Q4 vs Q2-Q3	1.59	1.52	1.68	**<0.001**
Age	1.04	1.04	1.05	**<0.001**
EF < 40%	1.22	1.17	1.28	**<0.001**
SPAP > 40	1.20	1.15	1.26	**<0.001**
GFR < 30	1.38	1.29	1.48	**<0.001**
BMI > 30	0.81	0.77	0.86	**<0.001**
AF	0.91	0.87	0.95	**<0.001**
IHD	0.93	0.89	0.97	**0.001**
HTN	0.86	0.82	0.91	**<0.001**
CVA	1.10	1.05	1.15	**<0.001**
COPD	1.22	1.15	1.29	**<0.001**
GDMT	1.02	0.97	1.07	0.404
Anemia	1.48	1.41	1.55	**<0.001**
DM	0.95	0.91	0.99	**0.018**
Baseline UA	1.08	1.06	1.11	**<0.001**

Table S12. Multivariable Cox Model of Mortality in Patients Without Oncological Disease: *Adjusted associations after excluding patients with a history of oncological disease.*

Variable	HR	CI Lower	CI Upper	p-value
≤Q1 vs Q2-Q3	0.81	0.76	0.85	**<0.001**
≥Q4 vs Q2-Q3	1.63	1.55	1.71	**<0.001**
Age	1.05	1.04	1.05	**<0.001**
EF < 40%	1.23	1.17	1.28	**<0.001**
SPAP > 40	1.19	1.14	1.24	**<0.001**
GFR < 30	1.38	1.30	1.47	**<0.001**
BMI > 30	0.82	0.78	0.87	**<0.001**
AF	0.91	0.87	0.95	**<0.001**
IHD	0.91	0.88	0.95	**<0.001**
HTN	0.84	0.80	0.88	**<0.001**
CVA	1.12	1.07	1.17	**<0.001**
GDMT	1.05	1.00	1.11	**0.036**
Anemia	1.50	1.43	1.56	**<0.001**
DM	0.95	0.91	0.99	**0.024**

Baseline UA	1.10	1.08	1.13	<0.001

Table S13. Multivariable Cox Model of Mortality in Patients Without COPD: *Adjusted associations after excluding patients with chronic obstructive pulmonary disease.*

**Variable**	**HR**	**CI Lower**	**CI Upper**	**p-value**
≤Q1 vs Q2-Q3	0.78	0.73	0.83	**<0.001**
≥Q4 vs Q2-Q3	1.56	1.48	1.65	**<0.001**
Age	1.05	1.04	1.05	**<0.001**
EF < 40%	1.23	1.17	1.29	**<0.001**
SPAP > 40	1.22	1.16	1.28	**<0.001**
GFR < 30	1.43	1.33	1.54	**<0.001**
BMI > 30	0.84	0.79	0.89	**<0.001**
AF	0.90	0.86	0.95	**<0.001**
IHD	0.89	0.85	0.94	**<0.001**
HTN	0.88	0.83	0.93	**<0.001**
CVA	1.13	1.07	1.19	**<0.001**
COPD	1.21	1.14	1.29	**<0.001**
GDMT	1.03	0.98	1.09	0.246
Anemia	1.52	1.44	1.60	**<0.001**
DM	0.99	0.95	1.04	0.800
Baseline UA	1.09	1.06	1.12	**<0.001**

Table S14. Multivariable Cox Model of Mortality in Male Patients: *Adjusted associations between uric acid variability and mortality among male patients only.*

**Variable**	**HR**	**CI Lower**	**CI Upper**	**p-value**
≤Q1 vs Q2-Q3	0.81	0.75	0.87	**<0.001**
≥Q4 vs Q2-Q3	1.65	1.54	1.76	**<0.001**
Age	1.04	1.04	1.05	**<0.001**
EF < 40%	1.17	1.09	1.25	**<0.001**
SPAP > 40	1.20	1.13	1.28	**<0.001**
GFR < 30	1.31	1.19	1.43	**<0.001**
BMI > 30	0.79	0.74	0.85	**<0.001**
AF	0.89	0.84	0.95	**<0.001**
IHD	0.94	0.88	1.00	**0.034**
HTN	0.84	0.78	0.90	**<0.001**
CVA	1.06	0.99	1.13	0.079
COPD	1.19	1.10	1.29	**<0.001**
GDMT	1.04	0.97	1.12	0.227

Anemia	1.35	1.27	1.44	**<0.001**
DM	0.91	0.85	0.96	**0.001**
Baseline UA	1.11	1.08	1.15	**<0.001**

Table S15. Multivariable Cox Model of Mortality in Female Patients: *Adjusted associations between uric acid variability and mortality among female patients only.*

**Variable**	**HR**	**CI Lower**	**CI Upper**	**p-value**
≤Q1 vs Q2-Q3	0.87	0.81	0.94	**<0.001**
≥Q4 vs Q2-Q3	1.51	1.41	1.61	**<0.001**
Age	1.06	1.05	1.06	**<0.001**
EF < 40%	1.33	1.25	1.41	**<0.001**
SPAP > 40	1.11	1.04	1.17	**0.001**
GFR < 30	1.29	1.18	1.40	**<0.001**
BMI > 30	0.84	0.77	0.91	**<0.001**
AF	0.93	0.87	0.98	**0.009**
IHD	0.90	0.85	0.96	**<0.001**
HTN	0.89	0.83	0.95	**<0.001**
CVA	1.00	0.94	1.07	0.908
COPD	1.10	1.01	1.18	**0.020**
GDMT	1.06	0.99	1.13	0.090
Anemia	1.32	1.24	1.41	**<0.001**
DM	0.85	0.80	0.90	**<0.001**
Baseline UA	1.15	1.11	1.18	**<0.001**

Table S16. Multivariable Cox Model of Mortality in Octogenarians: *Adjusted associations between uric acid variability and mortality among patients aged ≥80 years.*

**Variable**	**HR**	**CI Lower**	**CI Upper**	**p-value**
≤Q1 vs Q2-Q3	0.74	0.69	0.79	**<0.001**
≥Q4 vs Q2-Q3	1.65	1.56	1.75	**<0.001**
Age	1.04	1.03	1.04	**<0.001**
EF < 40%	1.14	1.08	1.20	**<0.001**
SPAP > 40	1.28	1.22	1.35	**<0.001**
GFR < 30	1.42	1.32	1.53	**<0.001**
BMI > 30	0.80	0.76	0.85	**<0.001**
AF	0.89	0.85	0.94	**<0.001**
IHD	0.93	0.88	0.97	**0.003**
HTN	0.85	0.80	0.90	**<0.001**
CVA	1.17	1.10	1.23	**<0.001**

COPD	1.30	1.23	1.38	**<0.001**
GDMT	1.03	0.97	1.09	0.286
Anemia	1.54	1.46	1.62	**<0.001**
DM	1.05	1.00	1.10	0.069
Baseline UA	1.07	1.04	1.10	**<0.001**

Table S17. Multivariable Cox Model of Mortality in Non-Octogenarians: *Adjusted associations between uric acid variability and mortality among patients aged <80 years.*

Variable	HR	CI Lower	CI Upper	p-value
≤Q1 vs Q2-Q3	0.78	0.73	0.82	**<0.001**
≥Q4 vs Q2-Q3	1.68	1.59	1.77	**<0.001**
Age	1.05	1.05	1.05	**<0.001**
EF < 40%	1.14	1.08	1.19	**<0.001**
SPAP > 40	1.14	1.09	1.20	**<0.001**
BMI > 30	0.84	0.80	0.89	**<0.001**
AF	0.89	0.85	0.93	**<0.001**
IHD	0.88	0.84	0.92	**<0.001**
HTN	0.88	0.83	0.92	**<0.001**
CVA	1.10	1.05	1.15	**<0.001**
COPD	1.23	1.16	1.30	**<0.001**
GDMT	1.03	0.98	1.09	0.206
Anemia	1.41	1.34	1.47	**<0.001**
DM	1.00	0.95	1.04	0.845
Baseline UA	1.08	1.05	1.10	**<0.001**

Table S18. Multivariable Cox Model of Mortality in Patients Without CKD: *Adjusted associations between uric acid variability and mortality after excluding patients with chronic kidney disease.*

Variable	HR	CI Lower	CI Upper	p-value
≤Q1 vs Q2-Q3	0.95	0.84	1.07	0.375
≥Q4 vs Q2-Q3	1.10	0.97	1.26	0.125
Age	1.04	1.03	1.04	**<0.001**
EF < 40%	1.34	1.20	1.50	**<0.001**
SPAP > 40	1.22	1.10	1.35	**<0.001**
BMI > 30	0.94	0.84	1.07	0.354
AF	0.93	0.84	1.03	0.183
IHD	0.97	0.87	1.07	0.514
HTN	0.86	0.75	0.98	**0.025**
CVA	0.96	0.86	1.08	0.502

COPD	1.07	0.94	1.22	0.302
GDMT	1.09	0.96	1.22	0.187
Anemia	1.23	1.06	1.44	**0.008**
DM	1.03	0.92	1.14	0.647
Baseline UA	1.02	0.96	1.08	0.588

Table S19. Multivariable Cox Model of Mortality in Patients With CKD: *Adjusted associations between uric acid variability and mortality restricted to patients with chronic kidney disease.*

**Variable**	**HR**	**CI Lower**	**CI Upper**	**p-value**
≤Q1 vs Q2-Q3	0.78	0.71	0.86	**<0.001**
≥Q4 vs Q2-Q3	1.60	1.47	1.74	**<0.001**
Age	1.04	1.04	1.05	**<0.001**
EF < 40%	1.14	1.06	1.24	**0.001**
SPAP > 40	1.20	1.11	1.29	**<0.001**
GFR < 30	1.38	1.23	1.55	**<0.001**
BMI > 30	0.78	0.71	0.87	**<0.001**
AF	0.90	0.83	0.97	**0.005**
IHD	0.91	0.85	0.98	**0.015**
HTN	0.85	0.78	0.92	**<0.001**
CVA	1.12	1.03	1.22	**0.006**
COPD	1.18	1.07	1.30	**0.001**
GDMT	1.03	0.95	1.12	0.490
Anemia	1.45	1.33	1.57	**<0.001**
DM	1.02	0.95	1.10	0.594
Baseline UA	1.11	1.07	1.16	**<0.001**

Table S20. Multivariable Cox Model of Mortality in Patients Not Receiving Furosemide: *Adjusted associations between uric acid variability and mortality among patients not treated with furosemide.*

**Variable**	**HR**	**CI Lower**	**CI Upper**	**p-value**
≤Q1 vs Q2-Q3	0.80	0.75	0.85	**<0.001**
≥Q4 vs Q2-Q3	1.55	1.47	1.64	**<0.001**
Age	1.05	1.04	1.05	**<0.001**
EF < 40%	1.25	1.19	1.31	**<0.001**
SPAP > 40	1.22	1.17	1.28	**<0.001**
GFR < 30	1.40	1.30	1.50	**<0.001**
BMI > 30	0.82	0.77	0.87	**<0.001**
AF	0.90	0.86	0.95	**<0.001**
IHD	0.91	0.87	0.96	**<0.001**

HTN	0.84	0.80	0.89	**<0.001**
CVA	1.10	1.04	1.16	**<0.001**
COPD	1.20	1.14	1.27	**<0.001**
GDMT	0.99	0.92	1.07	0.816
Anemia	1.46	1.39	1.53	**<0.001**
DM	0.93	0.89	0.97	**0.002**
Baseline UA	1.11	1.08	1.13	**<0.001**

Table S21. Multivariable Cox Model of Mortality in Patients Receiving Furosemide: *Adjusted associations between uric acid variability and mortality among patients treated with furosemide.*

Variable	HR	CI Lower	CI Upper	p-value
≤Q1 vs Q2-Q3	1.07	0.74	1.56	0.715
≥Q4 vs Q2-Q3	1.49	1.04	2.14	**0.030**
Age	2.42	1.75	3.35	**<0.001**
EF < 40%	0.83	0.61	1.14	0.247
SPAP > 40	1.18	0.86	1.63	0.296
GFR < 30	1.48	0.93	2.35	0.100
BMI > 30	0.73	0.51	1.05	0.091
AF	0.78	0.57	1.08	0.131
IHD	1.05	0.77	1.44	0.758
HTN	1.05	0.74	1.50	0.780
CVA	1.09	0.78	1.52	0.606
COPD	1.09	0.73	1.62	0.668
GDMT	0.92	0.59	1.43	0.704
Anemia	1.05	0.77	1.43	0.763
DM	0.92	0.68	1.26	0.620
Baseline UA	1.07	0.74	1.56	0.715

Table S22. Multivariable Cox Model of Mortality in Patients Receiving UA Lowering Drugs: *Adjusted associations between uric acid variability and mortality among patients treated with UA lowering drugs.*

Variable	HR	CI Lower	CI Upper	p-value
≤Q1 vs Q2-Q3	0.71	0.52	0.98	0.038

≥Q4 vs Q2-Q3	1.38	1.05	1.81	0.021
Age	1.63	1.27	2.09	0.000
EF < 40%	0.80	0.63	1.02	0.073
SPAP > 40	1.46	1.14	1.88	0.003
GFR < 30	2.26	1.65	3.09	0.000
BMI > 30	0.78	0.59	1.03	0.085
AF	1.25	0.98	1.60	0.074
IHD	1.04	0.82	1.32	0.764
HTN	0.95	0.73	1.23	0.693
CVA	1.10	0.85	1.43	0.473
COPD	1.20	0.83	1.73	0.327
GDMT	1.05	0.76	1.47	0.761
Anemia	1.06	0.84	1.35	0.613
DM	1.13	0.88	1.45	0.340
TAPSE	0.96	0.87	1.05	0.362
UA Baseline Value	0.97	0.92	1.03	0.344

Table S23. Multivariable Cox Model Adjusted for TAPSE: *Adjusted associations between uric acid variability and mortality adjusted for TAPSE.*

UA Trajectories - Mortality & Outcomes

Contrast	HR and CI	p-value
Q1: Descending	0.78 (0.69-0.88)	**<0.001**
Q1: Ascending	1.10 (0.98-1.23)	0.077
Q2-Q3: Descending	0.92 (0.86-0.98)	**0.023**
Q2-Q3: Ascending	1.52 (1.43-1.62)	**<0.001**
Q4: Descending	0.97 (0.88-1.06)	0.507
Q4: Ascending	1.78 (1.64-1.93)	**<0.001**

Table S24. Unadjusted Associations Between Uric Acid Trajectories and Outcomes Across Variability Strata: *Unadjusted Cox models of Q1 and Q4 trajectory groups compared with the reference (Q2-Q3), stratified by baseline uric acid variability (≤Q1, Q2-Q3, ≥Q4).*

Variable	HR	CI Lower	CI Upper	p-value
≤Q1 vs Q2-Q3	0.67	0.59	0.76	**<0.001**
≥Q4 vs Q2-Q3	0.94	0.84	1.06	0.292
Age	1.06	1.06	1.06	**<0.001**
EF < 40%	1.16	1.06	1.27	**0.001**
SPAP > 40	1.26	1.15	1.37	**<0.001**
GFR < 30	1.52	1.30	1.79	**<0.001**
BMI > 30	0.75	0.67	0.83	**<0.001**
AF	0.94	0.86	1.02	0.132
IHD	0.92	0.85	1.00	**0.046**
HTN	0.85	0.78	0.93	**<0.001**
CVA	1.12	1.02	1.23	**0.020**
COPD	1.27	1.13	1.42	**<0.001**
GDMT	1.07	0.97	1.18	0.176
Anemia	1.57	1.44	1.72	**<0.001**
DM	0.99	0.91	1.07	0.762

Baseline UA	1.38	1.31	1.45	**<0.001**

Table S25. Adjusted Cox Model of Uric Acid Trajectories and Outcomes in Patients With Low Variability (≤Q1): *Fully adjusted model examining the association of UA trajectory groups with outcomes among patients in the lowest variability stratum.*

Variable	HR	CI Lower	CI Upper	p-value
≤Q1 vs Q2-Q3	0.85	0.79	0.91	**<0.001**
≥Q4 vs Q2-Q3	1.27	1.20	1.35	**<0.001**
Age	1.04	1.04	1.05	**<0.001**
EF < 40%	1.25	1.18	1.32	**<0.001**
SPAP > 40	1.22	1.15	1.28	**<0.001**
GFR < 30	1.61	1.48	1.75	**<0.001**
BMI > 30	0.81	0.76	0.87	**<0.001**
AF	0.90	0.85	0.95	**<0.001**
IHD	0.88	0.84	0.93	**<0.001**
HTN	0.85	0.80	0.91	**<0.001**
CVA	1.13	1.06	1.20	**<0.001**
COPD	1.24	1.16	1.33	**<0.001**
GDMT	1.06	0.99	1.12	0.091
Anemia	1.46	1.38	1.54	**<0.001**
DM	0.97	0.92	1.02	0.253
Baseline UA	1.18	1.14	1.22	**<0.001**

Table S26. Adjusted Cox Model of Uric Acid Trajectories and Outcomes in Patients With Moderate Variability (Q2-Q3): *Adjusted associations of UA trajectory groups with outcomes among patients with moderate variability.*

Variable	HR	CI Lower	CI Upper	p-value
≤Q1 vs Q2-Q3	0.85	0.77	0.94	**0.001**
≥Q4 vs Q2-Q3	1.71	1.57	1.86	**<0.001**
Age	1.03	1.03	1.03	**<0.001**
EF < 40%	1.13	1.05	1.21	**0.001**
SPAP > 40	1.08	1.01	1.15	**0.031**
GFR < 30	1.24	1.14	1.36	**<0.001**
BMI > 30	0.83	0.76	0.90	**<0.001**
AF	0.82	0.77	0.88	**<0.001**

IHD	0.95	0.88	1.01	0.101
HTN	0.85	0.78	0.92	**<0.001**
CVA	1.01	0.94	1.09	0.701
COPD	1.06	0.98	1.15	0.152
GDMT	1.03	0.95	1.11	0.527
Anemia	1.26	1.17	1.36	**<0.001**
DM	0.90	0.84	0.97	**0.003**
Baseline UA	1.14	1.10	1.18	**<0.001**

Table S27. Adjusted Cox Model of Uric Acid Trajectories and Outcomes in Patients With High Variability (≥Q4): *Adjusted associations of UA trajectory groups with outcomes among patients in the highest variability stratum.*

PSM-Weighted Mortality subgroup models

Variable	HR	CI Lower	CI Upper	p-value
≤Q1 vs Q2-Q3	0.93	0.86	1.00	0.066
≥Q4 vs Q2-Q3	1.48	1.38	1.59	**<0.001**
Age	1.05	1.04	1.05	**<0.001**
EF < 40%	1.08	0.55	2.14	0.815
SPAP > 40	1.23	1.14	1.34	**<0.001**
GFR < 30	1.59	1.45	1.75	**<0.001**
BMI > 30	0.81	0.75	0.88	**<0.001**
AF	0.88	0.82	0.95	**0.001**
IHD	0.86	0.80	0.92	**<0.001**
HTN	0.79	0.71	0.87	**<0.001**
CVA	1.08	1.00	1.17	0.066
COPD	1.30	1.19	1.43	**<0.001**
GDMT	1.05	0.96	1.15	0.286
Anemia	1.42	1.31	1.55	**<0.001**
DM	1.08	0.99	1.17	0.068
Baseline UA	1.19	1.14	1.24	**<0.001**

Table S28. Multivariable PSM-Adjusted Cox Model of Mortality in Patients With HFrEF: *Adjusted PSM Cox regression for HFrEF patients, reporting treatment contrasts (Q1 and Q4 vs Q2-Q3) and clinical covariates.*

Variable	HR	CI Lower	CI Upper	p-value
≤Q1 vs Q2-Q3	0.82	0.75	0.89	**<0.001**
≥Q4 vs Q2-Q3	1.14	1.04	1.25	**0.004**
Age	1.01	1.01	1.01	**<0.001**
EF < 40%	1.12	0.58	2.16	0.727
SPAP > 40	1.10	1.00	1.22	0.053
GFR < 30	1.15	0.99	1.35	0.075
BMI > 30	0.85	0.77	0.93	**0.001**
AF	0.87	0.80	0.96	**0.003**
IHD	0.92	0.84	1.02	0.103
HTN	0.89	0.79	1.00	**0.049**
CVA	0.86	0.78	0.96	**0.005**
COPD	0.97	0.87	1.08	0.538
GDMT	0.93	0.83	1.04	0.208

Anemia	1.23	1.12	1.35	**<0.001**
DM	1.00	0.91	1.10	0.964
Baseline UA	1.03	0.97	1.09	0.341

Table S29. Multivariable PSM-Adjusted Cox Model of Mortality in Patients With HFpEF: *Adjusted PSM Cox regression for HFpEF patients, reporting treatment contrasts (Q1 and Q4 vs Q2-Q3) and clinical covariates.*

Variable	HR	CI Lower	CI Upper	p-value
≤Q1 vs Q2-Q3	0.86	0.81	0.92	**<0.001**
≥Q4 vs Q2-Q3	1.49	1.41	1.58	**<0.001**
Age	1.05	1.04	1.05	**<0.001**
EF < 40%	1.18	1.10	1.26	**<0.001**
SPAP > 40	1.23	1.16	1.31	**<0.001**
GFR < 30	1.53	1.41	1.67	**<0.001**
BMI > 30	0.80	0.74	0.86	**<0.001**
AF	0.93	0.88	0.99	0.030
IHD	0.95	0.89	1.01	0.103
HTN	0.85	0.79	0.92	**<0.001**
CVA	1.13	1.05	1.20	**<0.001**
COPD	1.25	1.15	1.35	**<0.001**
GDMT	1.05	0.98	1.14	0.173
Anemia	1.58	1.48	1.69	**<0.001**
DM	0.97	0.91	1.04	0.405
Baseline UA	1.23	1.18	1.27	**<0.001**

Table S30. Multivariable PSM-Adjusted Cox Model of Mortality in Patients Without Oncological Disease: *Adjusted PSM Cox regression excluding oncological patients.*

Variable	HR	CI Lower	CI Upper	p-value
≤Q1 vs Q2-Q3	0.84	0.79	0.89	**<0.001**
≥Q4 vs Q2-Q3	1.51	1.43	1.59	**<0.001**
Age	1.05	1.05	1.05	**<0.001**
EF < 40%	1.17	1.10	1.24	**<0.001**
SPAP > 40	1.24	1.16	1.31	**<0.001**
GFR < 30	1.58	1.46	1.70	**<0.001**
BMI > 30	0.78	0.73	0.84	**<0.001**
AF	0.92	0.86	0.97	**0.004**
IHD	0.95	0.90	1.01	0.107
HTN	0.85	0.79	0.91	**<0.001**
CVA	1.16	1.08	1.23	**<0.001**

GDMT	1.11	1.03	1.19	**0.004**
Anemia	1.57	1.48	1.67	**<0.001**
DM	1.01	0.96	1.08	0.623
Baseline UA	1.24	1.20	1.29	**<0.001**

Table S31. Multivariable PSM-Adjusted Cox Model of Mortality in Patients Without COPD: *Adjusted PSM Cox regression excluding COPD patients*

**Variable**	**HR**	**CI Lower**	**CI Upper**	**p-value**
≤Q1 vs Q2-Q3	0.89	0.82	0.95	**<0.001**
≥Q4 vs Q2-Q3	1.56	1.47	1.66	**<0.001**
Age	1.04	1.04	1.05	**<0.001**
EF < 40%	1.16	1.08	1.25	**<0.001**
SPAP > 40	1.29	1.20	1.39	**<0.001**
GFR < 30	1.60	1.46	1.76	**<0.001**
BMI > 30	0.82	0.76	0.89	**<0.001**
AF	0.89	0.83	0.95	**<0.001**
IHD	1.00	0.93	1.07	0.930
HTN	0.91	0.84	0.98	**0.020**
CVA	1.13	1.05	1.22	**<0.001**
COPD	1.35	1.24	1.47	**<0.001**
GDMT	1.05	0.96	1.14	0.304
Anemia	1.60	1.48	1.72	**<0.001**
DM	1.02	0.95	1.09	0.674
Baseline UA	1.22	1.18	1.27	**<0.001**

Table S32. Multivariable PSM-Adjusted Cox Model of Mortality in Male Patients: *Adjusted PSM Cox regression restricted to males.*

Variable	HR	CI Lower	CI Upper	p-value
≤Q1 vs Q2-Q3	0.80	0.74	0.86	**<0.001**
≥Q4 vs Q2-Q3	1.46	1.37	1.55	**<0.001**
Age	1.05	1.04	1.05	**<0.001**
EF < 40%	1.13	1.04	1.23	**<0.001**
SPAP > 40	1.33	1.23	1.43	**<0.001**
GFR < 30	1.51	1.36	1.67	**<0.001**
BMI > 30	0.77	0.70	0.85	**<0.001**
AF	0.92	0.86	1.00	0.123
IHD	1.09	1.01	1.18	*0.030*
HTN	0.87	0.80	0.94	**<0.001**
CVA	1.16	1.07	1.26	**<0.001**
COPD	1.27	1.15	1.40	**<0.001**
GDMT	1.17	1.07	1.28	**<0.001**

Anemia	1.67	1.55	1.81	**<0.001**
DM	1.01	0.94	1.09	0.723
Baseline UA	1.25	1.19	1.30	**<0.001**

Table S33. Multivariable PSM-Adjusted Cox Model of Mortality in Female Patients: *Adjusted PSM Cox regression restricted to females.*

**Variable**	**HR**	**CI Lower**	**CI Upper**	**p-value**
≤Q1 vs Q2-Q3	0.81	0.74	0.88	**<0.001**
≥Q4 vs Q2-Q3	1.29	1.19	1.39	**<0.001**
Age	1.05	1.04	1.06	**<0.001**
EF < 40%	1.32	1.21	1.45	**<0.001**
SPAP > 40	1.15	1.06	1.24	**0.001**
GFR < 30	1.32	1.19	1.48	**<0.001**
BMI > 30	0.80	0.72	0.90	**<0.001**
AF	1.00	0.92	1.09	0.989
IHD	0.93	0.86	1.01	0.087
HTN	0.93	0.84	1.04	0.189
CVA	1.02	0.93	1.11	0.731
COPD	1.19	1.07	1.32	**0.001**
GDMT	1.07	0.97	1.18	0.176
Anemia	1.30	1.19	1.42	**<0.001**
DM	0.87	0.80	0.94	**<0.001**
Baseline UA	1.24	1.19	1.30	**<0.001**

Table S34. Multivariable PSM-Adjusted Cox Model of Mortality in Octogenarians: *Adjusted PSM Cox regression restricted to patients aged ≥80 years.*

**Variable**	**HR**	**CI Lower**	**CI Upper**	**p-value**
≤Q1 vs Q2-Q3	0.86	0.80	0.92	**<0.001**
≥Q4 vs Q2-Q3	1.69	1.59	1.80	**<0.001**
Age	1.04	1.03	1.04	**<0.001**
EF < 40%	1.04	0.97	1.12	0.302
SPAP > 40	1.36	1.26	1.46	**<0.001**
GFR < 30	1.65	1.51	1.80	**<0.001**
BMI > 30	0.78	0.72	0.84	**<0.001**
AF	0.89	0.83	0.95	**<0.001**
IHD	1.04	0.97	1.12	0.264
HTN	0.84	0.77	0.90	**<0.001**
CVA	1.20	1.12	1.30	**<0.001**
COPD	1.35	1.25	1.47	**<0.001**
GDMT	1.08	0.99	1.17	0.091

Anemia	1.68	1.56	1.80	**<0.001**
DM	1.07	1.00	1.16	0.283
Baseline UA	1.23	1.18	1.27	**<0.001**

Table S35. Multivariable PSM-Adjusted Cox Model of Mortality in Patients Younger Than 80 Years: *Adjusted PSM Cox regression restricted to patients <80 years.*

Variable	HR	CI Lower	CI Upper	p-value
≤Q1 vs Q2-Q3	0.67	0.56	0.79	**<0.001**
≥Q4 vs Q2-Q3	1.22	1.05	1.40	**0.012**
Age	1.04	1.03	1.05	**<0.001**
EF < 40%	1.20	1.01	1.44	**0.041**
SPAP > 40	1.36	1.16	1.58	**<0.001**
BMI > 30	0.91	0.78	1.08	0.292
AF	0.91	0.77	1.06	0.231
IHD	1.09	0.93	1.27	0.300
HTN	0.84	0.69	1.04	0.110
CVA	1.00	0.84	1.18	0.972
COPD	1.24	1.04	1.48	**0.020**
GDMT	1.13	0.94	1.35	0.190
Anemia	1.19	0.97	1.46	0.091
DM	1.17	0.99	1.37	0.075
Baseline UA	1.15	1.04	1.27	**0.012**

Table S36. Multivariable PSM-Adjusted Cox Model of Mortality in Patients With CKD: *Adjusted PSM Cox regression restricted to patients with CKD.*

Variable	HR	CI Lower	CI Upper	p-value
≤Q1 vs Q2-Q3	0.89	0.83	0.95	**<0.001**
≥Q4 vs Q2-Q3	1.56	1.48	1.65	**<0.001**
Age	1.05	1.05	1.06	**<0.001**
EF < 40%	1.11	1.04	1.18	**<0.001**
SPAP > 40	1.24	1.16	1.33	**<0.001**
BMI > 30	0.81	0.76	0.88	**<0.001**
AF	0.93	0.87	0.99	0.021
IHD	0.95	0.89	1.02	0.143
HTN	0.88	0.82	0.95	**<0.001**
CVA	1.14	1.06	1.22	**<0.001**
COPD	1.38	1.28	1.50	**<0.001**
GDMT	1.11	1.03	1.19	0.010

Anemia	1.48	1.39	1.58	**<0.001**
DM	1.08	1.01	1.15	0.020
Baseline UA	1.24	1.20	1.28	**<0.001**

Table S37. Multivariable PSM-Adjusted Cox Model of Mortality in Patients Without CKD: *Adjusted PSM Cox regression restricted to patients without CKD.*

Variable	HR	CI Lower	CI Upper	p-value
≤Q1 vs Q2-Q3	0.86	0.80	0.92	**<0.001**
≥Q4 vs Q2-Q3	1.45	1.37	1.54	**<0.001**
Age	1.05	1.05	1.05	**<0.001**
EF < 40%	1.23	1.14	1.32	**<0.001**
SPAP > 40	1.32	1.24	1.41	**<0.001**
GFR < 30	1.57	1.44	1.72	**<0.001**
BMI > 30	0.82	0.76	0.88	**<0.001**
AF	0.93	0.87	0.99	0.020
IHD	0.98	0.91	1.04	0.471
HTN	0.83	0.77	0.90	**<0.001**
CVA	1.11	1.03	1.19	**<0.001**
COPD	1.28	1.18	1.39	**<0.001**
GDMT	0.99	0.89	1.10	0.803
Anemia	1.58	1.47	1.69	**<0.001**
DM	0.95	0.89	1.02	0.142
Baseline UA	1.22	1.17	1.26	**<0.001**

Table S38. Multivariable PSM-Adjusted Cox Model of Mortality in Patients Receiving Furosemide: *Adjusted PSM Cox regression restricted to patients on furosemide.*

Variable	HR	CI Lower	CI Upper	p-value
≤Q1 vs Q2-Q3	0.91	0.82	1.01	0.091
≥Q4 vs Q2-Q3	1.53	1.38	1.69	**<0.001**
Age	1.05	1.04	1.05	**<0.001**
EF < 40%	1.00	0.89	1.12	0.981
SPAP > 40	1.29	1.16	1.44	**<0.001**
GFR < 30	1.59	1.37	1.85	**<0.001**
BMI > 30	0.73	0.65	0.83	**<0.001**
AF	0.91	0.82	1.01	0.091
IHD	0.96	0.86	1.07	0.453
HTN	0.88	0.78	1.00	0.096
CVA	1.21	1.07	1.36	**<0.001**

COPD	1.29	1.12	1.48	**<0.001**
GDMT	1.06	0.94	1.18	0.350
Anemia	1.70	1.51	1.90	**<0.001**
DM	1.02	0.92	1.14	0.672
Baseline UA	1.23	1.16	1.31	**<0.001**

Table S39. Multivariable PSM-Adjusted Cox Model of Mortality in Patients Not Receiving Furosemide: *Adjusted PSM Cox regression restricted to patients not on furosemide.*

Variable	HR	CI Lower	CI Upper	p-value
≤Q1 vs Q2-Q3	0.68	0.55	0.84	**<0.001**
≥Q4 vs Q2-Q3	1.45	1.21	1.74	**<0.001**
Age	1.05	1.03	1.07	**<0.001**
EF < 40%	1.24	0.99	1.55	0.057
SPAP > 40	1.26	1.01	1.57	0.038
GFR < 30	1.47	1.11	1.94	0.006
BMI > 30	0.66	0.52	0.84	0.001
AF	0.83	0.67	1.01	0.067
IHD	1.00	0.81	1.24	0.991
HTN	0.62	0.48	0.79	**<0.001**
CVA	1.09	0.85	1.42	0.493
COPD	1.13	0.83	1.56	0.437
GDMT	1.23	0.91	1.67	0.179
Anemia	1.80	1.43	2.26	**<0.001**
DM	1.03	0.83	1.28	0.803
Baseline UA	1.02	0.96	1.07	0.590

Table S40. Multivariable PSM-Adjusted Cox Model of Mortality in Patients Receiving Allopurinol: *Adjusted PSM Cox regression restricted to patients not on allopurinol.*

HF related hospitalization subgroup and sensitivity analysis

Variable	HR	CI Lower	CI Upper	p-value
≤Q1 vs Q2-Q3	0.89	0.81	0.97	**0.012**
≥Q4 vs Q2-Q3	1.16	1.05	1.28	**0.002**
Age	1.00	0.99	1.00	0.190
SPAP > 40	1.19	1.10	1.29	**<0.001**
GFR < 30	1.04	0.92	1.17	0.537
BMI > 30	1.05	0.95	1.16	0.318
AF	0.92	0.85	0.99	**0.031**
IHD	1.03	0.95	1.12	0.425
HTN	0.92	0.85	1.01	0.068
CVA	0.93	0.85	1.01	0.104
COPD	0.92	0.83	1.02	0.119
GDMT	1.03	0.94	1.13	0.580
Anemia	1.15	1.06	1.25	**0.001**
DM	1.01	0.93	1.09	0.820
Baseline UA	1.17	1.12	1.22	**<0.001**

Table S41 . Multivariable Cox Model of HF-Related Hospitalization in HFrEF Patients:
Adjusted associations between uric acid variability and HF-related hospitalization among patients with reduced ejection fraction.

Variable	HR	CI Lower	CI Upper	p-value
≤Q1 vs Q2-Q3	0.80	0.74	0.87	**<0.001**
≥Q4 vs Q2-Q3	1.17	1.08	1.27	**<0.001**
Age	1.01	1.01	1.01	**<0.001**
SPAP > 40	1.06	1.00	1.14	0.068
GFR < 30	1.11	1.00	1.22	**0.045**
BMI > 30	0.91	0.85	0.98	**0.012**
AF	0.92	0.86	0.98	**0.011**
IHD	0.96	0.90	1.03	0.242
HTN	0.86	0.79	0.93	**<0.001**
CVA	0.91	0.85	0.98	0.015
COPD	0.99	0.92	1.07	0.793
GDMT	0.96	0.89	1.04	0.284
Anemia	1.19	1.11	1.28	**<0.001**
DM	0.98	0.92	1.05	0.565
Baseline UA	1.01	0.98	1.05	0.438

Table S42. Multivariable Cox Model of HF-Related Hospitalization in HFpEF Patients: *Adjusted associations between uric acid variability and HF-related hospitalization among patients with preserved ejection fraction.*

Variable	HR	CI Lower	CI Upper	p-value
≤Q1 vs Q2-Q3	0.85	0.80	0.92	**<0.001**
≥Q4 vs Q2-Q3	1.22	1.14	1.31	**<0.001**
Age	1.00	1.00	1.00	0.103
EF < 40%	1.36	1.27	1.45	**<0.001**
SPAP > 40	1.11	1.05	1.18	**0.001**
GFR < 30	1.07	0.98	1.17	0.121
BMI > 30	0.94	0.88	1.01	0.076
AF	0.95	0.89	1.00	0.060
IHD	1.00	0.94	1.06	0.954
HTN	0.88	0.83	0.95	**<0.001**
CVA	0.89	0.84	0.95	**<0.001**
COPD	0.95	0.88	1.02	0.125
GDMT	0.98	0.91	1.05	0.538
Anemia	1.15	1.09	1.23	**<0.001**
DM	1.00	0.94	1.06	0.877
Baseline UA	1.09	1.06	1.12	**<0.001**

Table S43. Multivariable Cox Model of HF-Related Hospitalization in Patients Without Oncological Disease: *Adjusted associations after excluding patients with a history of oncological disease.*

Variable	HR	CI Lower	CI Upper	p-value
≤Q1 vs Q2-Q3	0.86	0.80	0.92	**<0.001**
≥Q4 vs Q2-Q3	1.19	1.11	1.28	**<0.001**
Age	1.00	1.00	1.00	0.092
EF < 40%	1.35	1.27	1.44	<0.001
SPAP > 40	1.11	1.04	1.17	**0.001**
GFR < 30	1.08	0.99	1.18	0.067
BMI > 30	0.96	0.90	1.02	0.205
AF	0.92	0.87	0.98	**0.006**
IHD	0.99	0.94	1.05	0.844
HTN	0.86	0.81	0.92	**<0.001**
CVA	0.88	0.83	0.94	**<0.001**
GDMT	0.98	0.92	1.05	0.648
Anemia	1.15	1.08	1.22	**<0.001**
DM	1.00	0.94	1.06	0.972

Baseline UA	1.10	1.06	1.13	**<0.001**

Table S44. Multivariable Cox Model of HF-Related Hospitalization in Patients Without COPD: *Adjusted associations after excluding patients with chronic obstructive pulmonary disease.*

Variable	HR	CI Lower	CI Upper	p-value
≤Q1 vs Q2-Q3	0.92	0.85	0.99	**0.035**
≥Q4 vs Q2-Q3	1.20	1.10	1.30	**<0.001**
Age	1.00	1.00	1.00	0.699
EF < 40%	1.32	1.23	1.42	**<0.001**
SPAP > 40	1.15	1.08	1.24	**<0.001**
GFR < 30	1.12	1.02	1.24	**0.023**
BMI > 30	0.94	0.86	1.01	0.106
AF	0.92	0.86	0.99	**0.019**
IHD	0.96	0.89	1.03	0.231
HTN	0.87	0.81	0.94	**<0.001**
CVA	0.90	0.84	0.97	**0.008**
COPD	0.93	0.85	1.01	0.066
GDMT	0.99	0.91	1.07	0.799
Anemia	1.16	1.08	1.25	**<0.001**
DM	0.99	0.92	1.06	0.701
Baseline UA	1.12	1.08	1.16	**<0.001**

Table S45. Multivariable Cox Model of HF-Related Hospitalization in Male Patients: *Adjusted associations between uric acid variability and HF-related hospitalization among male patients only.*

Variable	HR	CI Lower	CI Upper	p-value
≤Q1 vs Q2-Q3	0.77	0.70	0.84	**<0.001**
≥Q4 vs Q2-Q3	1.16	1.06	1.27	**0.002**
Age	1.01	1.00	1.01	**<0.001**
EF < 40%	1.32	1.20	1.45	**<0.001**
SPAP > 40	1.09	1.01	1.18	**0.025**
GFR < 30	0.99	0.88	1.12	0.915
BMI > 30	0.96	0.88	1.05	0.403
AF	0.92	0.85	1.00	**0.039**
IHD	1.02	0.95	1.11	0.556
HTN	0.88	0.80	0.96	**0.006**
CVA	0.95	0.87	1.03	0.204
COPD	0.97	0.88	1.07	0.566

GDMT	0.97	0.89	1.06	0.555
Anemia	1.16	1.07	1.26	**<0.001**
DM	0.99	0.92	1.07	0.813
Baseline UA	1.07	1.02	1.11	**0.003**

Table S46. Multivariable Cox Model of HF-Related Hospitalization in Female Patients: *Adjusted associations between uric acid variability and HF-related hospitalization among female patients only.*

Variable	HR	CI Lower	CI Upper	p-value
≤Q1 vs Q2-Q3	0.78	0.71	0.86	**<0.001**
≥Q4 vs Q2-Q3	1.16	1.05	1.28	**0.003**
Age	1.02	1.01	1.03	**<0.001**
EF < 40%	1.21	1.10	1.32	**<0.001**
SPAP > 40	1.09	1.00	1.18	**0.042**
GFR < 30	1.17	1.04	1.31	**0.010**
BMI > 30	0.95	0.86	1.06	0.366
AF	0.93	0.85	1.01	0.068
IHD	1.08	1.00	1.17	0.065
HTN	1.01	0.91	1.11	0.916
CVA	0.92	0.84	1.00	0.052
COPD	0.99	0.90	1.10	0.864
GDMT	0.96	0.87	1.05	0.352
Anemia	1.20	1.10	1.30	**<0.001**
DM	1.01	0.94	1.10	0.709
Baseline UA	1.05	1.01	1.10	**0.017**

Table S47. Multivariable Cox Model of HF-Related Hospitalization in Octogenarians: *Adjusted associations between uric acid variability and HF-related hospitalization among patients aged ≥80 years.*

Variable	HR	CI Lower	CI Upper	p-value
≤Q1 vs Q2-Q3	0.88	0.82	0.96	**0.002**
≥Q4 vs Q2-Q3	1.20	1.11	1.30	**<0.001**
Age	1.00	1.00	1.00	0.623
EF < 40%	1.43	1.33	1.54	**<0.001**
SPAP > 40	1.14	1.07	1.22	**<0.001**
GFR < 30	1.02	0.93	1.13	0.651
BMI > 30	0.95	0.89	1.02	0.155
AF	0.94	0.88	1.00	0.060
IHD	0.98	0.91	1.04	0.472

HTN	0.84	0.77	0.90	***<0.001***
CVA	0.91	0.84	0.98	***0.010***
COPD	0.94	0.87	1.01	0.102
GDMT	1.01	0.93	1.09	0.836
Anemia	1.17	1.09	1.25	**<0.001**
DM	0.98	0.92	1.06	0.655
Baseline UA	1.12	1.08	1.16	**<0.001**

Table S48. Multivariable Cox Model of HF-Related Hospitalization in Non-Octogenarians: *Adjusted associations between uric acid variability and HF-related hospitalization among patients aged <80 years.*

Variable	HR	CI Lower	CI Upper	p-value
≤Q1 vs Q2-Q3	0.80	0.75	0.86	**<0.001**
≥Q4 vs Q2-Q3	1.16	1.08	1.24	**<0.001**
Age	1.00	1.00	1.00	0.087
EF < 40%	1.33	1.24	1.42	**<0.001**
SPAP > 40	1.11	1.05	1.18	**<0.001**
BMI > 30	0.97	0.91	1.04	0.370
AF	0.92	0.87	0.98	**0.006**
IHD	0.98	0.92	1.03	0.420
HTN	0.89	0.83	0.95	**<0.001**
CVA	0.91	0.85	0.96	**0.002**
COPD	0.96	0.90	1.03	0.285
GDMT	0.97	0.90	1.03	0.326
Anemia	1.16	1.10	1.23	**<0.001**
DM	1.00	0.94	1.06	0.970
Baseline UA	1.09	1.06	1.13	**<0.001**

Table S49. Multivariable Cox Model of HF-Related Hospitalization in Patients Without CKD: *Adjusted associations after excluding patients with chronic kidney disease.*

Variable	HR	CI Lower	CI Upper	p-value
≤Q1 vs Q2-Q3	0.93	0.79	1.10	0.410
≥Q4 vs Q2-Q3	1.23	1.02	1.47	**0.026**
Age	1.01	1.00	1.01	**0.028**
EF < 40%	1.44	1.23	1.70	**<0.001**
SPAP > 40	1.10	0.95	1.27	0.187
GFR < 30	1.04	0.14	7.58	0.970
BMI > 30	0.98	0.84	1.15	0.785
AF	1.07	0.93	1.23	0.346

IHD	1.08	0.94	1.24	0.297
HTN	0.84	0.69	1.03	0.095
CVA	0.98	0.85	1.14	0.843
COPD	0.90	0.76	1.08	0.249
GDMT	1.01	0.86	1.19	0.894
Anemia	1.23	0.97	1.56	0.086
DM	1.12	0.96	1.31	0.136
Baseline UA	1.04	0.96	1.12	0.386

Table S50. Multivariable Cox Model of HF-Related Hospitalization in Patients With CKD: *Adjusted associations restricted to patients with chronic kidney disease.*

Variable	HR	CI Lower	CI Upper	p-value
≤Q1 vs Q2-Q3	0.86	0.80	0.93	**<0.001**
≥Q4 vs Q2-Q3	1.19	1.10	1.28	**<0.001**
Age	1.00	1.00	1.01	**0.001**
EF < 40%	1.36	1.27	1.45	**<0.001**
SPAP > 40	1.09	1.02	1.16	**0.007**
GFR < 30	1.00	0.91	1.10	0.983
BMI > 30	0.98	0.91	1.05	0.521
AF	0.92	0.86	0.98	**0.006**
IHD	1.03	0.96	1.09	0.402
HTN	0.87	0.81	0.94	**<0.001**
CVA	0.94	0.88	1.01	0.099
COPD	0.95	0.88	1.02	0.178
GDMT	0.91	0.82	1.00	0.061
Anemia	1.22	1.14	1.30	**<0.001**
DM	1.00	0.94	1.06	0.966
Baseline UA	1.10	1.06	1.14	**<0.001**

Table S51. Multivariable Cox Model of HF-Related Hospitalization in Patients Receiving Furosemide: *Adjusted associations between uric acid variability and HF-related hospitalization among patients treated with furosemide.*

Variable	HR	CI Lower	CI Upper	p-value
≤Q1 vs Q2-Q3	0.91	0.80	1.02	0.115
≥Q4 vs Q2-Q3	1.24	1.09	1.40	**0.001**
Age	1.00	0.99	1.00	0.267
EF < 40%	1.40	1.25	1.56	**<0.001**
SPAP > 40	1.17	1.06	1.30	**0.003**
GFR < 30	1.28	1.10	1.50	**0.002**

BMI > 30	0.83	0.74	0.93	**0.002**
AF	0.96	0.87	1.06	0.434
IHD	0.90	0.81	0.99	**0.037**
HTN	0.90	0.80	1.02	0.096
CVA	0.83	0.75	0.93	**0.001**
COPD	0.99	0.87	1.12	0.854
GDMT	1.01	0.91	1.13	0.812
Anemia	1.07	0.97	1.19	0.185
DM	1.01	0.91	1.12	0.847
Baseline UA	1.08	1.02	1.13	**0.006**

Table S52. Multivariable Cox Model of HF-Related Hospitalization in Patients Not Receiving Furosemide: *Adjusted associations between uric acid variability and HF-related hospitalization among patients not treated with furosemide.*

Variable	HR	CI Lower	CI Upper	p-value
≤Q1 vs Q2-Q3	1.30	0.75	2.26	0.354
≥Q4 vs Q2-Q3	1.16	0.66	2.02	0.605
Age	1.06	0.64	1.75	0.830
EF < 40%	0.75	0.45	1.25	0.274
SPAP > 40	1.13	0.71	1.82	0.605
GFR < 30	1.04	0.54	1.99	0.908
BMI > 30	1.12	0.70	1.80	0.640
AF	1.43	0.88	2.34	0.148
IHD	1.39	0.85	2.27	0.192
HTN	1.73	1.01	2.96	0.045
CVA	0.95	0.55	1.62	0.847
COPD	2.12	0.97	4.66	0.061
GDMT	0.93	0.50	1.72	0.811
Anemia	2.19	1.28	3.72	0.004
DM	0.85	0.53	1.38	0.519
Baseline UA	1.02	0.80	1.31	0.844

Table S53. Multivariable Cox Model of HF-Related Hospitalization in Patients Receiving UA Lowering Drugs: *Adjusted associations between uric acid variability and HF-related hospitalization among patients treated with UA lowering drugs.*

Variable	HR	CI Lower	CI Upper	p-value
≤Q1 vs Q2-Q3	0.71	0.52	0.98	0.038
≥Q4 vs Q2-Q3	1.38	1.05	1.81	0.021
Age	1.63	1.27	2.09	0.000
EF < 40%	0.80	0.63	1.02	0.073
SPAP > 40	1.46	1.14	1.88	0.003
GFR < 30	2.26	1.65	3.09	0.000
BMI > 30	0.78	0.59	1.03	0.085
AF	1.25	0.98	1.60	0.074
IHD	1.04	0.82	1.32	0.764
HTN	0.95	0.73	1.23	0.693
CVA	1.10	0.85	1.43	0.473
COPD	1.20	0.83	1.73	0.327
GDMT	1.05	0.76	1.47	0.761
Anemia	1.06	0.84	1.35	0.613
DM	1.13	0.88	1.45	0.340
TAPSE	0.96	0.87	1.05	0.362
UA Baseline Value	0.97	0.92	1.03	0.344

Table S54. Multivariable Cox Model Adjusted for TAPSE: Adjusted associations between uric acid variability and HF Hospitalization adjusted for TAPSE.

Contrast	HR and CI	p-value
Q1: Descending	0.85 (0.75-0.97)	**0.023**
Q1: Ascending	0.61 (0.53-0.71)	**<0.001**
Q2-Q3: Descending	0.91 (0.84-0.99)	**0.040**
Q2-Q3: Ascending	0.88 (0.81-0.96)	**0.006**
Q4: Descending	1.15 (1.02-1.31)	**0.022**
Q4: Ascending	1.04 (0.92-1.17)	0.510

Table S55. Unadjusted Associations Between Uric Acid Trajectories and HF-Related Hospitalization Across Variability Strata: *Unadjusted Cox models comparing Q1 and Q4 UA-trajectory groups with the reference (Q2-Q3) within each baseline variability stratum (≤Q1, Q2-Q3, ≥Q4); HRs with 95% CIs and p-values are reported.*

Variable	HR	CI Lower	CI Upper	p-value
≤Q1 vs Q2-Q3	0.73	0.63	0.84	**<0.001**
≥Q4 vs Q2-Q3	0.63	0.54	0.73	**<0.001**
Age	1.01	1.00	1.01	**0.015**
EF < 40%	1.17	1.04	1.31	**0.010**
SPAP > 40	1.25	1.13	1.39	**<0.001**
GFR < 30	1.11	0.91	1.35	0.313
BMI > 30	0.81	0.72	0.91	**0.001**
AF	0.85	0.76	0.94	**0.001**
IHD	0.99	0.89	1.09	0.790
HTN	0.85	0.76	0.96	**0.008**
CVA	0.86	0.77	0.97	**0.014**
COPD	0.90	0.78	1.03	0.114
GDMT	1.09	0.96	1.23	0.177
Anemia	1.09	0.98	1.20	0.114
DM	1.01	0.91	1.12	0.850
Baseline UA	1.23	1.15	1.31	**<0.001**

Table S56. Adjusted Cox Model of HF-Related Hospitalization in Patients With Low UA Variability (≤Q1): *Fully adjusted Cox regression assessing UA-trajectory groups (Q1 and Q4 vs Q2-Q3) among patients in the lowest variability stratum.*

Variable	HR	CI Lower	CI Upper	p-value
≤Q1 vs Q2-Q3	0.90	0.82	0.98	**0.016**
≥Q4 vs Q2-Q3	0.87	0.79	0.95	**0.001**
Age	1.00	1.00	1.01	**0.028**
EF < 40%	1.39	1.28	1.50	**<0.001**
SPAP > 40	1.09	1.01	1.17	**0.022**
GFR < 30	1.04	0.94	1.15	0.454
BMI > 30	0.99	0.91	1.08	0.851
AF	0.96	0.89	1.03	0.249
IHD	1.01	0.94	1.09	0.740
HTN	0.90	0.82	0.98	**0.013**
CVA	0.93	0.86	1.01	0.080
COPD	1.00	0.92	1.09	0.945
GDMT	0.92	0.85	1.00	0.050
Anemia	1.23	1.14	1.33	**<0.001**
DM	0.98	0.91	1.06	0.668
Baseline UA	1.07	1.02	1.12	**0.004**

Table S57. Adjusted Cox Model of HF-Related Hospitalization in Patients With Moderate UA Variability (Q2-Q3): *Adjusted associations of UA-trajectory groups with HF hospitalization in the moderate variability stratum (Q1 and Q4 vs Q2-Q3).*

Variable	HR	CI Lower	CI Upper	p-value
≤Q1 vs Q2-Q3	0.98	0.86	1.13	0.829
≥Q4 vs Q2-Q3	1.06	0.93	1.19	0.388
Age	1.00	0.99	1.00	0.209
EF < 40%	1.41	1.26	1.57	**<0.001**
SPAP > 40	1.11	1.00	1.23	0.043
GFR < 30	1.06	0.92	1.22	0.404
BMI > 30	0.96	0.85	1.08	0.508
AF	0.99	0.89	1.10	0.824
IHD	1.05	0.95	1.16	0.340
HTN	0.87	0.77	0.98	**0.022**
CVA	0.90	0.80	1.00	0.057
COPD	0.90	0.80	1.01	0.074
GDMT	1.04	0.93	1.18	0.477
Anemia	1.11	0.99	1.24	0.070
DM	1.00	0.90	1.11	0.970
Baseline UA	1.10	1.04	1.16	**0.001**

Table S58. Adjusted Cox Model of HF-Related Hospitalization in Patients With High UA Variability (≥Q4): *Adjusted associations of UA-trajectory groups with HF hospitalization in the highest variability stratum (Q1 and Q4 vs Q2-Q3)*

PSM-weighted HF related hospitalization

Variable	HR	CI Lower	CI Upper	p-value
≤Q1 vs Q2-Q3	0.93	0.83	1.04	0.185
≥Q4 vs Q2-Q3	1.12	1.00	1.26	**0.043**
Age	1.00	1.00	1.01	0.388
EF < 40%	1.08	0.96	1.21	0.185
SPAP > 40	1.16	1.03	1.30	**0.011**
GFR < 30	1.22	1.00	1.48	**0.047**
BMI > 30	1.05	0.92	1.21	0.450
AF	0.89	0.79	0.99	**0.027**
IHD	0.99	0.89	1.11	0.916
HTN	0.85	0.76	0.96	**0.010**
CVA	0.87	0.77	0.98	**0.024**
COPD	0.92	0.80	1.07	0.286
GDMT	1.09	0.96	1.24	0.179
Anemia	1.14	1.02	1.27	**0.022**
DM	0.98	0.88	1.09	0.693
Baseline UA	1.19	1.12	1.27	**<0.001**

Table S59. Multivariable PSM-Adjusted Cox Model of HF-Related Hospitalization in HFrEF: *Subanalysis restricted to HFrEF. Fully adjusted PSM Cox regression including treatment contrasts and covariates.*

Variable	HR	CI Lower	CI Upper	p-value
≤Q1 vs Q2-Q3	0.82	0.75	0.89	**<0.001**
≥Q4 vs Q2-Q3	1.14	1.04	1.25	**0.004**
Age	1.01	1.01	1.01	**<0.001**
EF < 40%	1.12	0.58	2.16	0.727
SPAP > 40	1.10	1.00	1.22	0.053
GFR < 30	1.15	0.99	1.35	0.075
BMI > 30	0.85	0.77	0.93	**0.001**
AF	0.87	0.80	0.96	**0.003**
IHD	0.92	0.84	1.02	0.103
HTN	0.89	0.79	0.99	**0.049**
CVA	0.86	0.78	0.96	**0.005**
COPD	0.97	0.87	1.08	0.538
GDMT	0.93	0.83	1.04	0.208
Anemia	1.23	1.12	1.35	**<0.001**
DM	1.00	0.91	1.10	0.964

Baseline UA	1.03	0.97	1.09	0.341

Table S60. Multivariable PSM-Adjusted Cox Model of HF-Related Hospitalization in HFpEF: *Subanalysis restricted to HFpEF. Fully adjusted PSM Cox regression including treatment contrasts and covariates.*

Variable	HR	CI Lower	CI Upper	p-value
≤Q1 vs Q2-Q3	0.88	0.81	0.95	**0.002**
≥Q4 vs Q2-Q3	1.21	1.11	1.31	**<0.001**
Age	1.00	1.00	1.01	0.439
EF < 40%	1.37	1.23	1.52	**<0.001**
SPAP > 40	1.17	1.07	1.27	**<0.001**
GFR < 30	1.11	0.96	1.29	0.169
BMI > 30	0.91	0.83	1.00	0.055
AF	0.91	0.83	0.98	**0.020**
IHD	0.95	0.87	1.03	0.204
HTN	0.88	0.80	0.97	**0.010**
CVA	0.83	0.75	0.91	**<0.001**
COPD	0.92	0.83	1.02	0.128
GDMT	0.97	0.87	1.07	0.522
Anemia	1.11	1.02	1.20	**0.016**
DM	1.00	0.92	1.08	0.925
Baseline UA	1.09	1.03	1.14	**0.001**

Table S61. Multivariable PSM-Adjusted Cox Model of HF-Related Hospitalization in Patients Without Oncological Disease: *Subanalysis restricted to patients without oncological disease. Fully adjusted PSM Cox regression including treatment contrasts and covariates.*

Variable	HR	CI Lower	CI Upper	p-value
≤Q1 vs Q2-Q3	0.86	0.80	0.93	**<0.001**
≥Q4 vs Q2-Q3	1.20	1.11	1.30	**<0.001**
Age	1.00	1.01	1.01	**0.015**
EF < 40%	1.27	1.16	1.40	**<0.001**
SPAP > 40	1.09	1.01	1.19	**0.037**
GFR < 30	1.16	1.00	1.35	**0.047**
BMI > 30	0.95	0.87	1.04	0.262
AF	0.85	0.79	0.92	**<0.001**
IHD	0.96	0.88	1.04	0.300
HTN	0.86	0.79	0.94	**0.001**
CVA	0.86	0.79	0.94	**0.001**
GDMT	1.02	0.92	1.12	0.732
Anemia	1.14	1.05	1.24	**0.001**

DM	0.95	0.87	1.03	0.203
Baseline UA	1.14	1.09	1.20	**<0.001**

Table S62. Multivariable PSM-Adjusted Cox Model of HF-Related Hospitalization in Patients Without COPD: *Subanalysis restricted to patients without COPD. Fully adjusted PSM Cox regression including treatment contrasts and covariates.*

Variable	HR	CI Lower	CI Upper	p-value
≤Q1 vs Q2-Q3	0.95	0.86	1.04	0.261
≥Q4 vs Q2-Q3	1.19	1.09	1.31	**<0.001**
Age	1.00	1.00	1.01	0.347
EF < 40%	1.28	1.14	1.43	**<0.001**
SPAP > 40	1.18	1.06	1.31	**0.002**
GFR < 30	1.21	1.03	1.42	**0.020**
BMI > 30	0.97	0.86	1.09	0.586
AF	0.87	0.79	0.96	**0.006**
IHD	0.96	0.87	1.06	0.373
HTN	0.84	0.75	0.94	**0.003**
CVA	0.89	0.80	1.00	**0.044**
COPD	0.92	0.81	1.03	0.148
GDMT	0.93	0.83	1.05	0.228
Anemia	1.15	1.04	1.27	**0.006**
DM	0.97	0.88	1.08	0.620
Baseline UA	1.12	1.05	1.19	**<0.001**

Table S63. Multivariable PSM-Adjusted Cox Model of HF-Related Hospitalization in Male Patients: *Subanalysis restricted to male patients. Fully adjusted PSM Cox regression including treatment contrasts and covariates.*

Variable	HR	CI Lower	CI Upper	p-value
≤Q1 vs Q2-Q3	0.77	0.70	0.86	**<0.001**
≥Q4 vs Q2-Q3	1.12	1.01	1.24	**0.040**
Age	1.01	1.00	1.01	**0.003**
EF < 40%	1.25	1.07	1.46	**0.004**
SPAP > 40	1.12	1.01	1.25	**0.038**
GFR < 30	1.10	0.90	1.35	0.365
BMI > 30	0.90	0.81	1.01	0.071
AF	0.84	0.76	0.94	**0.002**
IHD	0.94	0.84	1.05	0.297
HTN	0.93	0.81	1.06	0.275
CVA	0.97	0.86	1.09	0.590
COPD	0.99	0.87	1.13	0.915

GDMT	1.04	0.91	1.19	0.577
Anemia	1.10	0.98	1.23	0.094
DM	0.97	0.87	1.09	0.622
Baseline UA	1.09	1.02	1.16	**0.013**

Table S64. Multivariable PSM-Adjusted Cox Model of HF-Related Hospitalization in Female Patients: *Subanalysis restricted to female patients. Fully adjusted PSM Cox regression including treatment contrasts and covariates.*

Variable	HR	CI Lower	CI Upper	p-value
≤Q1 vs Q2-Q3	0.79	0.71	0.88	**<0.001**
≥Q4 vs Q2-Q3	1.23	1.10	1.37	**<0.001**
Age	1.03	1.02	1.05	**<0.001**
EF < 40%	1.13	0.99	1.30	0.078
SPAP > 40	1.08	0.97	1.21	0.177
GFR < 30	1.18	0.98	1.41	0.080
BMI > 30	0.87	0.76	0.98	**0.025**
AF	0.90	0.81	1.01	0.067
IHD	1.08	0.96	1.20	0.199
HTN	1.03	0.90	1.18	0.643
CVA	0.87	0.77	0.97	**0.014**
COPD	1.03	0.90	1.18	0.690
GDMT	0.95	0.83	1.08	0.427
Anemia	1.24	1.11	1.39	**<0.001**
DM	1.03	0.92	1.15	0.582
Baseline UA	1.05	0.99	1.13	0.127

Table S65. Multivariable PSM-Adjusted Cox Model of HF-Related Hospitalization in Octogenarians: *Subanalysis restricted to octogenarians. Fully adjusted PSM Cox regression including treatment contrasts and covariates.*

Variable	HR	CI Lower	CI Upper	p-value
≤Q1 vs Q2-Q3	0.88	0.80	0.96	**0.004**
≥Q4 vs Q2-Q3	1.19	1.09	1.31	**<0.001**
Age	1.01	1.00	1.01	**0.039**
EF < 40%	1.38	1.23	1.55	**<0.001**
SPAP > 40	1.14	1.03	1.26	**0.011**
GFR < 30	1.18	1.00	1.39	0.052
BMI > 30	0.93	0.84	1.02	0.128
AF	0.90	0.82	0.99	**0.031**
IHD	0.96	0.87	1.06	0.409

HTN	0.79	0.71	0.88	**<0.001**
CVA	0.84	0.76	0.94	**0.002**
COPD	0.87	0.78	0.98	**0.024**
GDMT	1.01	0.89	1.13	0.926
Anemia	1.13	1.03	1.23	**0.010**
DM	0.94	0.85	1.05	0.274
Baseline UA	1.17	1.11	1.24	**<0.001**

Table S66. Multivariable PSM-Adjusted Cox Model of HF-Related Hospitalization in Patients <80 Years: *Subanalysis restricted to patients younger than 80 years. Fully adjusted PSM Cox regression including treatment contrasts and covariates.*

Variable	HR	CI Lower	CI Upper	p-value
≤Q1 vs Q2-Q3	0.83	0.76	0.90	**<0.001**
≥Q4 vs Q2-Q3	1.11	1.02	1.20	**0.015**
Age	1.00	1.00	1.01	**0.017**
EF < 40%	1.30	1.17	1.44	**<0.001**
SPAP > 40	1.10	1.01	1.20	**0.023**
BMI > 30	0.98	0.90	1.07	0.604
AF	0.90	0.83	0.97	**0.010**
IHD	0.95	0.87	1.03	0.182
HTN	0.87	0.79	0.95	**0.003**
CVA	0.89	0.81	0.97	**0.012**
COPD	0.94	0.85	1.03	0.192
GDMT	0.97	0.88	1.07	0.559
Anemia	1.13	1.05	1.23	**0.002**
DM	1.01	0.93	1.10	0.728
Baseline UA	1.10	1.05	1.15	**<0.001**

Table S67. Multivariable PSM-Adjusted Cox Model of HF-Related Hospitalization in Patients Without CKD: *Subanalysis restricted to patients without CKD. Fully adjusted PSM Cox regression including treatment contrasts and covariates.*

Variable	HR	CI Lower	CI Upper	p-value
≤Q1 vs Q2-Q3	0.94	0.79	1.13	0.532
≥Q4 vs Q2-Q3	1.17	0.98	1.41	0.088
Age	1.01	1.00	1.02	0.073
EF < 40%	1.48	1.14	1.91	**0.003**
SPAP > 40	1.14	0.92	1.40	0.229
BMI > 30	1.00	0.80	1.25	0.999

AF	0.95	0.76	1.18	0.639
IHD	0.99	0.80	1.21	0.902
HTN	0.95	0.72	1.27	0.748
CVA	1.02	0.83	1.26	0.838
COPD	1.02	0.79	1.32	0.854
GDMT	1.01	0.78	1.29	0.966
Anemia	1.29	0.94	1.78	0.114
DM	1.18	0.94	1.48	0.145
Baseline UA	1.05	0.93	1.19	0.438

Table S68. Multivariable PSM-Adjusted Cox Model of HF-Related Hospitalization in Patients With CKD: *Subanalysis restricted to patients with CKD. Fully adjusted PSM Cox regression including treatment contrasts and covariates.*

**Variable**	**HR**	**CI Lower**	**CI Upper**	**p-value**
≤Q1 vs Q2-Q3	0.84	0.73	0.97	**0.016**
≥Q4 vs Q2-Q3	1.19	1.03	1.36	**0.016**
Age	1.00	0.99	1.01	0.845
EF < 40%	1.52	1.27	1.83	**<0.001**
SPAP > 40	1.27	1.10	1.47	**0.001**
GFR < 30	1.63	1.27	2.08	**<0.001**
BMI > 30	0.82	0.70	0.96	**0.016**
AF	0.91	0.79	1.05	0.197
IHD	0.80	0.69	0.94	**0.005**
HTN	0.83	0.70	0.99	**0.042**
CVA	0.87	0.75	1.01	**0.048**
COPD	1.00	0.84	1.18	0.954
GDMT	1.07	0.91	1.26	0.429
Anemia	1.09	0.94	1.27	0.236
DM	1.07	0.92	1.26	0.370
Baseline UA	1.11	1.02	1.21	**0.014**

Table S69. Multivariable PSM-Adjusted Cox Model of HF-Related Hospitalization in Patients Not Receiving Furosemide: *Subanalysis restricted to patients not receiving furosemide. Fully adjusted PSM Cox regression including treatment contrasts and covariates.*

Variable	HR	CI Lower	CI Upper	p-value
≤Q1 vs Q2-Q3	0.87	0.80	0.95	**0.003**
≥Q4 vs Q2-Q3	1.17	1.07	1.27	**0.001**
Age	1.00	1.00	1.01	**0.020**
EF < 40%	1.29	1.15	1.44	**<0.001**

SPAP > 40	1.08	0.98	1.18	0.113
GFR < 30	1.04	0.89	1.21	0.613
BMI > 30	0.91	0.83	1.01	0.066
AF	0.84	0.77	0.91	**<0.001**
IHD	1.00	0.92	1.09	0.974
HTN	0.90	0.81	0.99	**0.041**
CVA	0.89	0.80	0.99	**0.027**
COPD	0.89	0.80	0.99	**0.043**
GDMT	0.84	0.72	0.97	**0.016**
Anemia	1.21	1.11	1.32	**<0.001**
DM	0.94	0.86	1.02	0.147
Baseline UA	1.15	1.09	1.21	**<0.001**

Table S70. Multivariable PSM-Adjusted Cox Model of HF-Related Hospitalization in Patients Receiving Furosemide*: Subanalysis restricted to patients receiving furosemide. Fully adjusted PSM Cox regression including treatment contrasts and covariates.*

Variable	HR	CI Lower	CI Upper	p-value
≤Q1 vs Q2-Q3	0.98	0.72	1.35	0.911
≥Q4 vs Q2-Q3	1.56	1.19	2.04	**0.001**
Age	1.00	0.98	1.02	0.933
EF < 40%	1.31	0.92	1.86	0.135
SPAP > 40	1.19	0.88	1.60	0.258
GFR < 30	1.15	0.83	1.61	0.401
BMI > 30	0.86	0.59	1.25	0.435
AF	0.89	0.65	1.20	0.442
IHD	0.82	0.60	1.13	0.227
HTN	1.14	0.78	1.65	0.505
CVA	0.82	0.59	1.13	0.220
COPD	0.93	0.62	1.40	0.740
GDMT	0.80	0.50	1.29	0.367
Anemia	1.04	0.77	1.40	0.807
DM	1.08	0.80	1.44	0.621
UAValue	1.07	0.98	1.16	0.122

Table S71. Multivariable PSM-Adjusted Cox Model of HF-Related Hospitalization in Patients Receiving Allopurinol*: Subanalysis restricted to patients receiving allopurinol. Fully adjusted PSM Cox regression including treatment contrasts and covariates.*

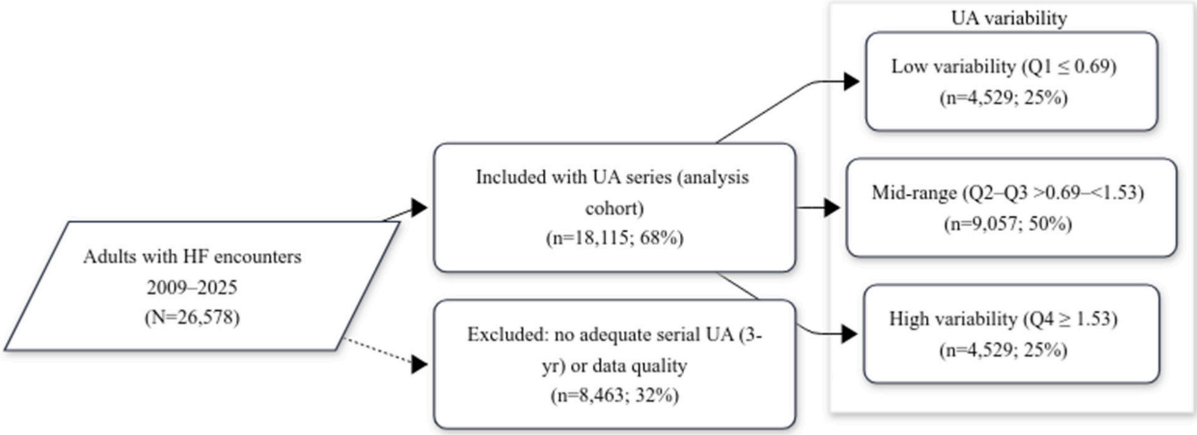

Figure S1. Study design - *Flowchart presenting exclusion and inclusion of patients alongside final groups.*

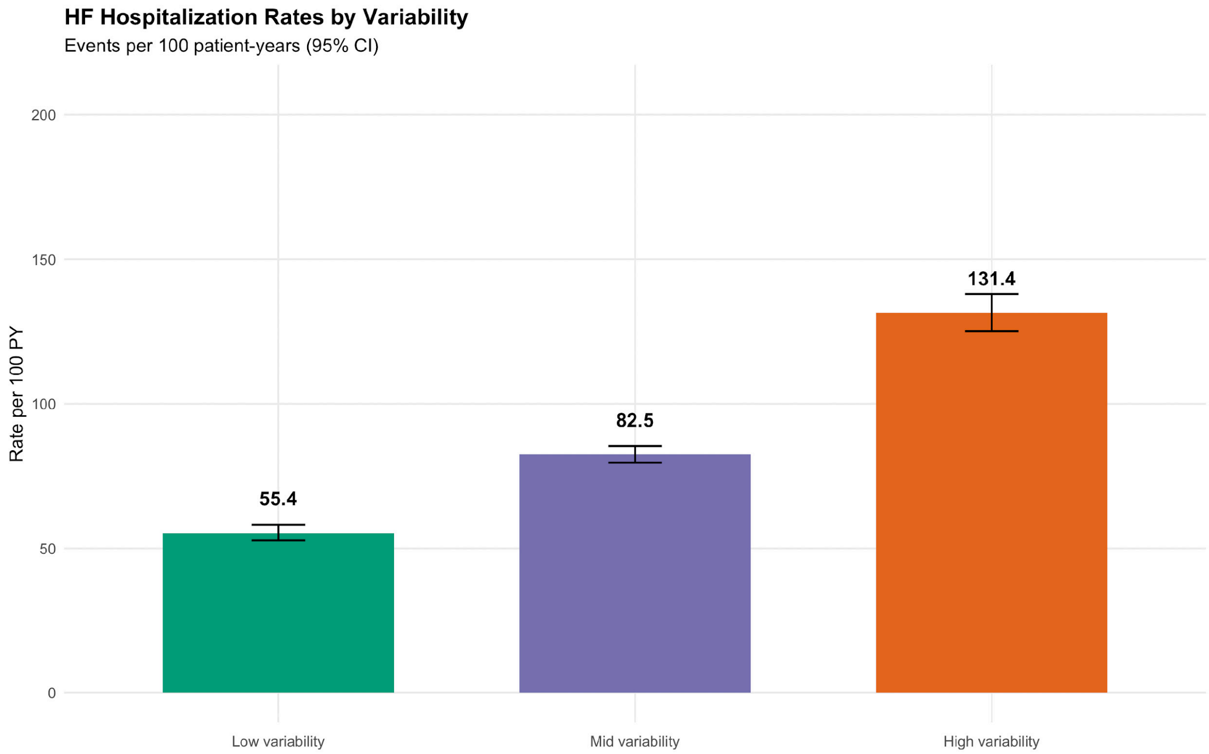

Figure S2. Rates of heart failure hospitalization per 100 patients - years by variability group (low, mid, high). Error bars indicate 95% confidence intervals.

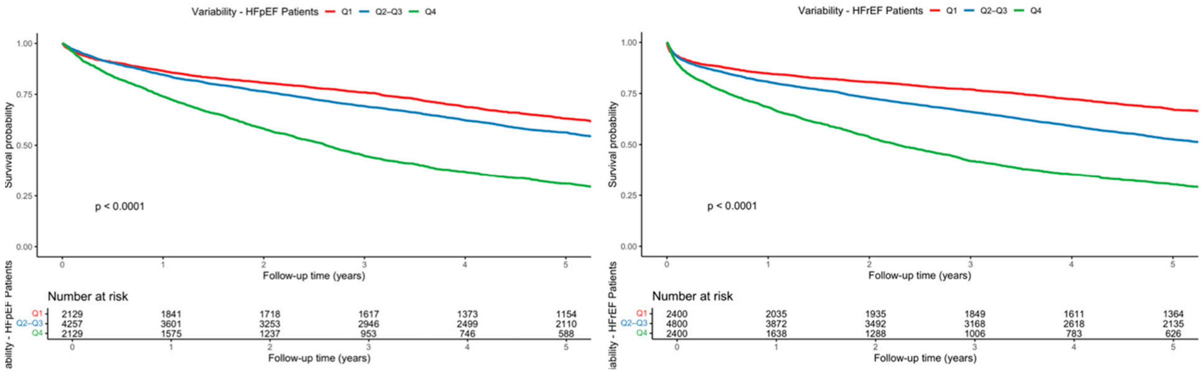

Figure S3: Survival by UA variability and heart failure subtype.

Kaplan–Meier curves for all-cause mortality stratified by UA variance in patients with HFrEF (right) and HFpEF (left). In both subtypes, low variability was associated with reduced mortality and high variability with increased mortality (log-rank p<0.0001).

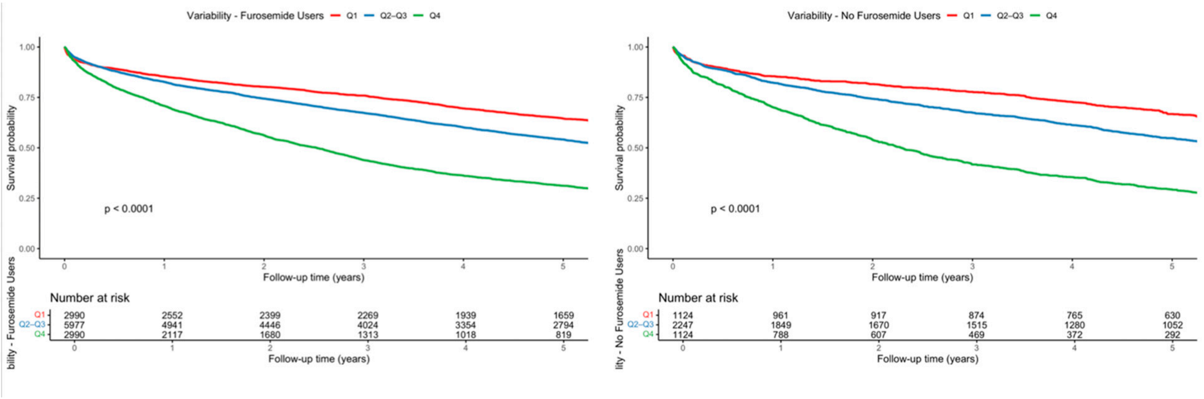

Figure S4: Survival by UA variability and furosemide use.

Kaplan–Meier curves for all-cause mortality stratified by UA variance in patients without (left) and with (right) furosemide use. In both strata, low variability was protective and high variability conferred increased risk (log-rank p<0.0001).
